# Assembly and Redox-Rich Hydride Chemistry of an Asymmetric Mo_2_S_2_ Platform

**DOI:** 10.3390/molecules25133090

**Published:** 2020-07-07

**Authors:** Alex McSkimming, Jordan W. Taylor, W. Hill Harman

**Affiliations:** Department of Chemistry, University of California, Riverside, California, CA 92521, USA; alexander.mcskimming@ucr.edu (A.M.); jtayl015@ucr.edu (J.W.T.)

**Keywords:** molybdenum sulfides, hydrides, redox-active ligands

## Abstract

Although molybdenum sulfide materials show promise as electrocatalysts for proton reduction, the hydrido species proposed as intermediates remain poorly characterized. We report herein the synthesis, reactions and spectroscopic properties of a molybdenum-hydride complex featuring an asymmetric Mo_2_S_2_ core. This molecule displays rich redox chemistry with electrochemical couples at *E*_½_ = −0.45, −0.78 and −1.99 V vs. Fc/Fc^+^. The corresponding hydrido-complexes for all three redox levels were isolated and characterized crystallographically. Through an analysis of solid-state bond metrics and DFT calculations, we show that the electron-transfer processes for the two more positive couples are centered predominantly on the pyridinediimine supporting ligand, whereas for the most negative couple electron-transfer is mostly Mo-localized.

## 1. Introduction

Efficient electro or photocatalytic reduction of protons to produce H_2_ gas remains an important challenge [1,2,3,4,5]. Not only is H_2_ a potential clean fuel, but is by far the most used chemical reductant [6]. H_2_ is typically produced industrially by the steam reforming of methane [7]. Although this process utilizes a nonrenewable hydrocarbon feedstock and generates large volumes of greenhouse gases, the low efficiency of current water splitting technologies makes steam reforming by far the most economical process [5]. As a result, a tremendous amount of research has revolved around discovery and optimization of new materials for the hydrogen evolution reaction (HER) [1,2,3,4,5]. Some of the most promising heterogeneous catalysts for this reaction are molybdenum sulfides (MoS*_x_*),which exhibit comparable overpotentials and current densities to Pd/Pt electrodes but are significantly cheaper [8,9,10]. Although the mechanism of proton reduction by MoS*_x_* materials is not fully understood, calculations implicate molybdenum-hydrido intermediates that are protonated to give H_2_ [11,12,13,14]. A number of molecular analogues for the surface interface of MoS*_x_* materials have been shown to be active HER catalysts, although Mo–H intermediates, if they are formed, have not been well-characterized [15,16,17,18,19,20,21,22]. Indeed, molecular Mo–H complexes featuring sulfur-donor ligands, particularly sulfides, are uncommon [23,24,25,26,27,28,29,30,31,32,33,34,35,36,37]. Consequently, we have been interested in preparing molybdenum-hydride complexes with sulfide ligands and studying their chemistry and spectroscopy. We report, herein, preparation of hydrido complexes featuring an Mo_2_S_2_ core and an examination of their redox properties, reactions and electronic structure.

## 2. Results

### 2.1. Synthesis of Complexes

In approaching this chemistry, we targeted Mo_2_S_2_ complexes for which each Mo site would reside in a distinct coordination environment. We hoped this would facilitate selective installation of a hydrido ligand at a single Mo, and so greatly simplify reactivity and spectroscopic studies. The Mo_2_S_2_ motif is common in the literature; however, all such complexes are approximately symmetric and, typically, coordinatively saturated [38].

**Scheme 1:**
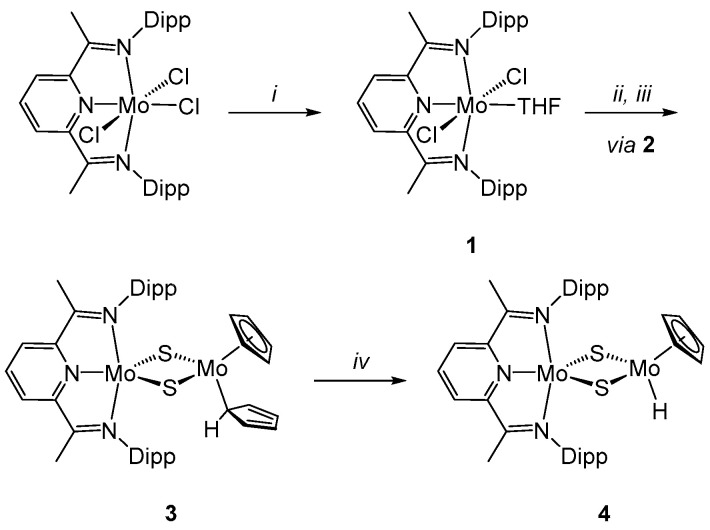
Synthesis of complexes **1**–**4**. Reagents and conditions: i. K(C_10_H_8_), THF (56%); ii. Cp_2_Mo(SH)_2_; iii. 2 eq. DBU (70% over 2 steps); iv. xs. NaBH_4_ (60%).

The assembly of an asymmetric Mo_2_S_2_ core and selective installation of a single hydride ligand are depicted in Scheme 1. First, reaction of the formally Mo^3+^ complex (PDI)MoCl_3_ [39] (PDI = (1,1′-(pyridine-2,6-diyl)bis(*N*-(2,6-diisopropylphenyl)ethan-1-imine)) with a stoichiometric amount of K(C_10_H_8_)(THF)_0.5_ in tetrahydrofuran (THF) gave the one-electron reduced complex trans-(PDI)MoCl_2_(THF) (**1** in Scheme 1) as a dark-green crystalline solid in 56% yield. Complex **1** was paramagnetic with an effective magnetic moment of 2.7 ± 0.2 μB in solution, consistent with an *S* = 1 ground state. Reaction of **1** with the dihydrosulfido complex Cp_2_Mo(SH)_2_ [40] gave the mono-hydrosulfido-bridged, trans-(PDI)MoCl_2_(μ-SH)Mo(SH)Cp_2_ (**2** in Scheme 1). This complex degraded rapidly in solution and so proved difficult to characterize. In spite of this, bond connectivity was assigned from a poor-quality single-crystal X-ray diffraction (XRD) study (Figure S1B). Deprotonation of **2** using 2 equivalents of 1,8-diazabicyclo [5.4.0]undec-7-ene (DBU) gave the sulfido-bridged complex (PDI)Mo(μ-S)_2_Mo(η^5^-Cp)(η^1^-Cp) (**3** in Scheme 1) as a dark-red solid. Strikingly, one Cp ligand switched from η^5^ to η^1^ coordination mode upon deprotonation. Although this complex could not be obtained in analytically pure form, it proved sufficiently pure to explore its utility as a precursor to Mo–H complexes.

Initially, we wondered whether protonolysis of **3** would yield CpH and the coordinately unsaturated cation, ((PDI)Mo(μ-S)_2_Mo(η^5^-Cp))^+^, which might then be reduced to give the hydrido complex (PDI)Mo(μ-S)_2_Mo(η^5^-Cp)(H) (**4** in Scheme 1). Unfortunately, attempted protonation of **3** using a variety of both weak and strong acids (e.g., (2,6-lutidinium) salts, H(OEt_2_)_2_[BAr^F^_4_] (BAr^F^_4_ = tetrakis(3,5-bistrifluoromethyl phenyl borate)) gave only complex mixtures. In further studies, however, we were surprised to find that reaction of **3** with strong one electron reductants such as KC_8_ gave mixtures displaying resonances < δ − 5 in crude ^1^H-NMR spectra, suggesting the formation of Mo-hydrido species. In an effort to isolate one such complex, **3** was reacted with an excess of Na[BH_4_] in THF to, fortuitously, produce the diamagnetic, yellow-brown hydrido complex **4** in 60% isolated yield. The corresponding deuterido complex **4**-D was synthesized analogously using Na[BD_4_] (95%-D). The ^1^H-NMR spectrum of **4** showed a signal of relative integration 1 at δ − 12.58, consistent with a metal-hydride functionality (see Figure S2A). As expected, the intensity of this peak was diminished by ca. 95% in the ^1^H-NMR spectrum of **4**-D. A 1D-^1^H-^1^H NOESY experiment with selective irradiation of the hydride resonance showed a strong cross-peak to the C_5_H_5_ signal, indicating that the hydrido ligand bound the Cp-substituted Mo (Figure S2B). Positive-mode liquid injected field desorption (LIFDI) mass spectrometry showed a peak at *m*/*z* 807.1475 (calcd. 807.1471) for the molecular ion, [**4**]^+^. As is not uncommon [41] the FT-IR spectrum of **4** contained no bands that could be unambiguously assigned to an Mo–H stretch (nor could any be identified in (Fourier transform infrared) FT-IR spectra of its reduced/oxidized congeners, vide infra; see Figures S3 and S4). Further, indirect evidence for the existence of a Mo–H moiety in **4** was provided by its rapid reaction with CDCl_3_ to produce CDHCl_2_ (see Figure S5) [42].

**Figure 1:**
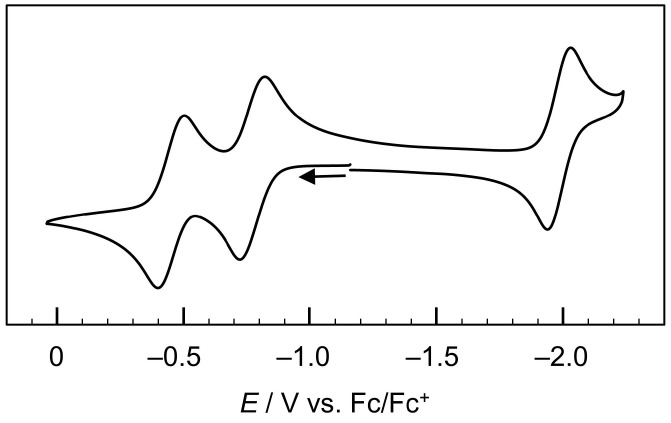
Cyclic voltammogram recorded for complex **4**. Conditions: 1 mM in THF–Na[BAr^F^_4_] at 100 mV s^−1^.

Given the potential relationship between Mo–H species and heterogenous MoS*_x_* HER catalysts (see Section 1), we were particularly interested to explore the redox chemistry of **4**. The cyclic voltammogram (CV) recorded for **4** in 0.1M Na[BAr^F^_4_] in THF is shown in Figure 1. On the CV timescale, complex **4** displayed rich redox chemistry, with reversible processes at *E*_½_ = −1.99, −0.78 and −0.45 V vs. Fc/Fc^+^ (open circuit potential ~−1.2 V; see Figure S6 for Randles-Sevcik plots). These were assigned to the **4**/[**4**]^−^, **4**/[**4**]^+^ and [**4**]^+^/[**4**]^2+^ couples, respectively. Given the anticipated extent of PDI-Mo orbital mixing (see also Section 2.4), [43] we expected that it would be problematic to assign these couples to localized electron-transfer processes from CV data alone. We wondered, then, if [**4**]^−^, [**4**]^+^ and/or [**4**]^2+^ could be isolated on a preparative scale and their properties examined to shed further light on the redox chemistry of these systems. 

**Scheme 2:**
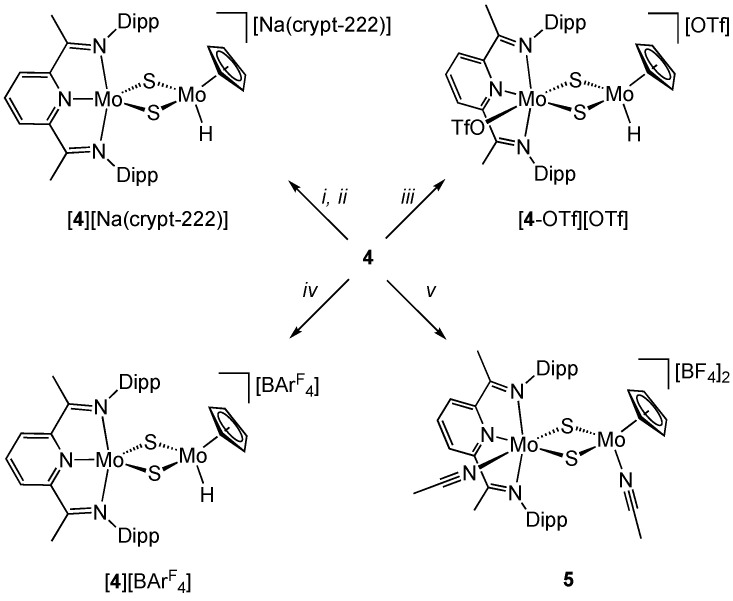
Redox and protonation chemistry of complex **4**. Reagents and conditions: i. xs. Na^0^; ii.1 Equation. [2.2.2]-Cryptand (crypt-222) (84% over 2 steps); iii. 2 Equation. Fc[OTf] (84%); iv. [Ph_3_C][BAr^F^_4_] (70%); v. xs. [Ph_2_NH_2_][BF_4_] (90%).

The redox and protonation chemistry of **4** is summarized in Scheme 2. First, reduction of **4** using metallic Na in diethyl ether followed by encapsulation of Na^+^ by [2.2.2]Cryptand (crypt-2.2.2) gave [**4**][Na(crypt-2.2.2)] as a brown, crystalline solid in 84% yield. Second, the dark-blue one-electron oxidized complex [**4**][BAr^F^_4_] was prepared from **4** in 70% yield by addition of 1 equivalent of either [Ph_3_C][BAr^F^_4_] or [Fc][BAr^F^_4_]. In the case of the former reagent, peaks for Gomberg’s dimer (the one-electron reduction product of Ph_3_C^+^) were observed in the ^1^H-NMR spectrum of the crude reaction mixture. In the absence of air and moisture, both the one-electron oxidized and reduced clusters were stable for prolonged periods (>1 week) at room temperature (RT) in both the solid state and in solution. Both had S = 1/2 ground-states as indicated by their electron paramagnetic resonance (EPR) spectra (see Section 2.2) and solution state magnetic moments of ~2 µB. 

In contrast to the syntheses of [**4**]^+/−^, isolation of the two-electron oxidized complex, [**4**]^2+^, proved more difficult. For example, addition of 2 equivalents of [Fc][BAr^F^_4_] to complex **4** in a variety of solvents gave intractable mixtures; the crude samples showed at least 3 diamagnetic Mo-H species. We speculated that the presumably highly electrophilic dication [**4**]^2+^ could be stabilized by incorporation of more coordinating anion(s). Accordingly, addition of 2 equivalents of [Fc][OTf] to **4** in THF cleanly generated the 2-electron oxidized, diamagnetic [(PDI)Mo(OTf)(μ-S)_2_Mo(η^5^-Cp)(H)][OTf] ([**4**-OTf][OTf]) as a red-brown crystalline solid in 84% yield. In the solid state one triflato group bound the PDI-ligated Mo with the other remaining outer sphere. In contrast to putative [**4**][BAr^F^_4_]_2_, ([**4**-OTf][OTf]) was indefinitely thermally stable in the solid state and decomposed only slowly over days in acetone solution at room temperature (RT). The ^1^H-NMR spectrum of [**4**-OTf][OTf] showed a broad signal at δ − 12.02 of relative integration 1 for the hydrido atom. Positive-mode LIFDI mass spectrometry performed on [**4**-(OTf)]+ showed a peak at *m*/*z* 956.1039 for the molecular ion [**4**-OTf]+ (calcd., 956.0991). 

In addition to its redox chemistry, we were also interested in examining the reaction of **4** with proton sources. Addition of excess [Ph_2_NH_2_][BF_4_] to a suspension of **4** in MeCN resulted in rapid evolution of H_2_ gas (by ^1^H-NMR spectroscopy) and formation of an intensely purple solution. From this mixture the dark-purple dication [(PDI)Mo(MeCN)(μ-S)_2_Mo(η^5-^Cp)(MeCN)][BF_4_]_2_ (**5**) was isolated in 90% yield. As with [**4**]^+/−^, complex **5** had an *S* = 1/2 ground-state as shown by EPR spectroscopy (see Section 2.2) and a solution state magnetic moment of 2.0 ± 0.1 μ_B_. In contrast to the neutral **4**, reaction of the anionic complex [**4**]^−^ with [Ph_2_NH_2_][BF_4_] gave a complex mixture of products. Repeated recrystallizations ultimately separated a small quantity of red-orange crystals, which were identified by XRD as a Mo_4_S_4_ cubane (**6**), which featured a partially hydrogenated PDI ligand (see Figure S1E). The variable protic stability of the Mo_2_S_2_ core, when in different formal redox states, will be an important consideration when optimizing these types of systems as catalysts for the HER. 

### 2.2. EPR Spectroscopy

**Figure 2:**
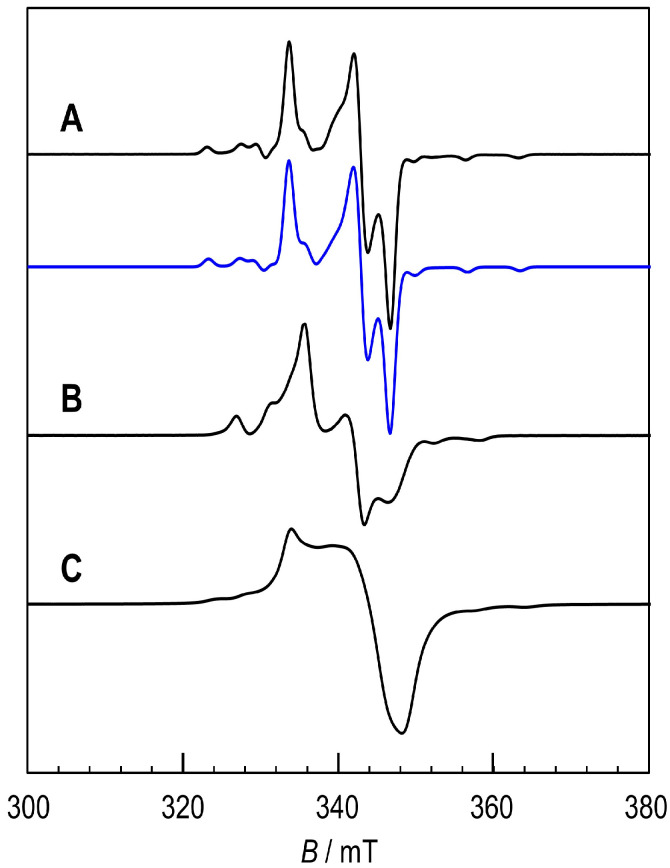
Continuous wave electron paramagnetic resonance (CW EPR) spectra recorded for [**4**]^+^ (A), [**4**]^−^ (B) and **5** (C) at 100 K, 9.32 GHz and 0.02 mW power. Experimental spectra are shown in black, simulations in blue. Conditions and (simulated) parameters: (A) 2-MeTHF; *g* = 2.00, 1.94, 1.92 (*g_iso_* = 1.95); H strain = 29, 36, 31 MHz; lw = 1 mT; (B) DMAc-2MeTHF (1:1); *g_iso_*~1.95 (C) MeCN-toluene (1:1); *g_iso_*~1.95.

As expected, the paramagnetic, half-integer spin [**4**]^+/−^ and **5** were all EPR-active. Continuous wave (CW) X-band EPR spectra recorded on dilute, frozen glasses of these complexes at 100 K showed complex envelopes of peaks with extensive hyperfine structure centered at g ≈ 2, indicative of an *S* = 1/2 ground state for all three species (Figure 2). The most likely origins of these hyperfine interactions are coupling to a combination of ^95/97^Mo, ^14^N in the PDI/MeCN ligand(s) and/or the hydrido ^1^H (for [**4**]^+/−^). The latter could be ruled out as the EPR spectra of [**4**]^+/−^ were indistinguishable from those recorded for [**4**–D]^+/−^. The EPR spectrum of the cation [**4**]^+^ (Figure 2A) was sufficiently resolved to be well-simulated by modelling hyperfine coupling to only natural abundance ^95/97^Mo (A = 110, 35, 180 MHz). For [**4**]^−^ and **5** (Figure 2B,C), however, conclusive simulations could not be obtained without overparameterization. 

### 2.3. Crystallography

**Figure 3:**
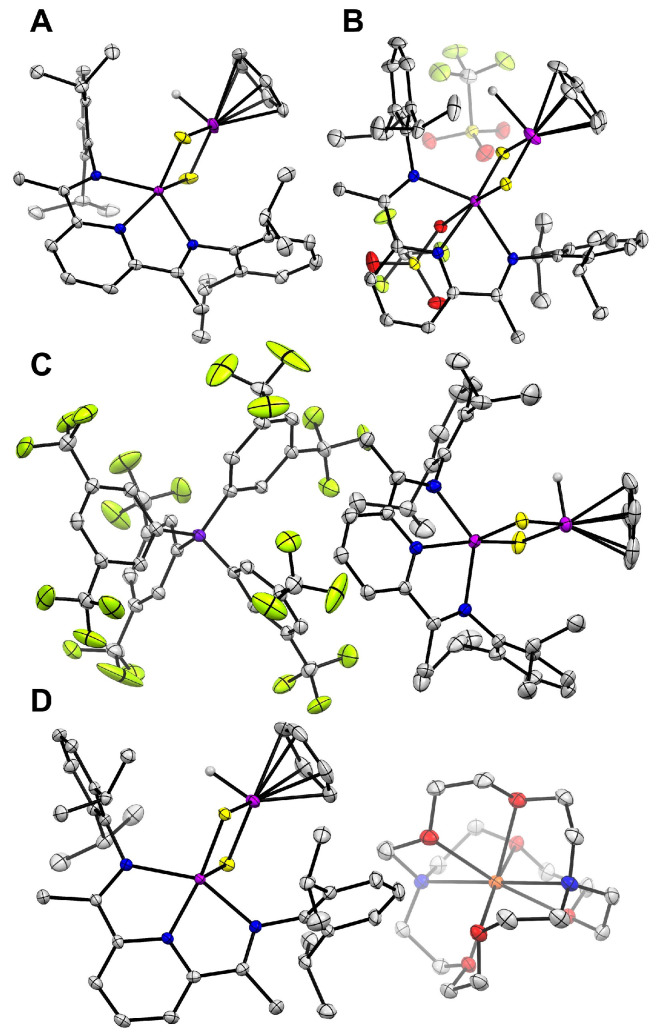
Thermal ellipsoid plots (50%) of (**A**) **4**, (B) [**4**-OTf][OTf] (**B**), (**C**) [**4**][BAr^F^_4_] and (**D**) [**4**][Na-crypt222]. Magenta, yellow, blue, orange, green and grey ellipsoids represent Mo, S, N, Na, F and C, respectively. Hydrogen atoms not bound to Mo and solvent molecules are omitted for clarity.

Single crystal XRD studies were performed on the four hydride complexes and also complexes **1**, **3** and **5**. Views from the crystal structures of the hydride complexes are presented in Figure 3; structures for **1**, **3**, **5** and connectivity assignments for **2** and **6** are shown in Figure S1. Selected experimental bond lengths and angles are summarized in Table 1. Electron density for the hydrido ligands was located in the difference map for all of the hydride complexes. 

PDIs are prototypical redox active ligands, and electron transfer processes for their complexes are frequently PDI-localized [44,45]. The extent to which the C = N_im_ (im = imine) and C_py_–C_im_ (py = pyridine) bonds lengthen and shorten, respectively, has proved a useful proxy for a qualitative assessment of the extent to which the PDI has been reduced. [46] PDI ligands are known to be able to accept up to 3 electrons to give the (formal) radical trianion, [PDI^•^]^3−^ [46]. With this in mind, inspection of the structural data revealed several key trends. First, the average Mo–S bond distances changed only slightly (≤0.01 Å) between [**4**-OTf]^+^, [**4**]^+^ and **4**. In contrast, for the anion [**4**]^−^, the Mo–S bonds at both Mo sites were longer than for the three more oxidized complexes by ~0.03 Å. In addition, upon one-electron reduction of **4** to [**4**]^−^, the Mo–N_im_ and Mo–N_py_ distances sharply increased and decreased, respectively, by ~0.05 Å. Second, the C = N_im_ and C_py_–C_im_ bonds expanded and contracted, respectively, on moving from [**4**-OTf]^+^ to [**4**]^+^ to **4** by roughly 0.03 Å per oxidation level. In contrast, metrics within the PDI system changed little between **4** and [**4**]^−^; indeed, the C_py_–C_im_ bonds lengthened very slightly. Overall, we hypothesise that upon sequential reduction of [**4**-OTf]^+^, the first two electrons are placed in MOs of mostly PDI-π* character, and the third into an MO of mostly metal character. This idea was explored further using computational methods (Section 2.4).

### 2.4. Density Functional Theory Calculations

**Figure 4:**
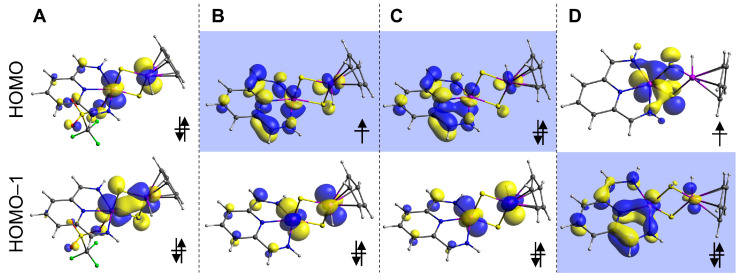
Selected unrestricted Kohn–Sham orbitals calculated for (**A**) [**4**-OTf]^+^, (**B**) [**4**]^+^, (**C**) **4**, and (**D**) [**4**]^−^. Molecular orbitals with a shaded background have predominantly PDI(π*) character.

In order to more thoroughly understand the electronic structure of these molecules, we turned to density functional theory (DFT). All calculations were performed on truncated structures obtained from XRD coordinates with the diisopropylphenyl (Dipp) and methyl groups of the PDI ligand replaced with protons. The unrestricted Kohn-Sham HOMOs and HOMO–1s calculated for the four hydride complexes are shown in Figure 4. The calculated orbital picture agrees well with the electronic structure assignments suggested by the solid state bond metrics. First, for [**4**-OTf]^+^ the doubly occupied HOMO is of predominantly Mo(d)-character and is essentially nonbonding with respect to the entire σ framework. The low-lying PDI-π* MO (not shown) remains unfilled. For complex [**4**]^+^, the singly-occupied HOMO is strongly localized (65%) on the PDI ligand with some π*-Mo(d) mixing. The orbital picture upon one-electron reduction to [**4**]^−^ remains similar with the HOMO now doubly-occupied. In contrast, the singly-occupied HOMO for the anion [**4**]^−^ is of predominantly (PDI)Mo(d)-character (54%). This MO is largely nonbonding with respect to the PDI ligand with minor Mo–N_im_(σ*) contributions. This would account for these bonds being slightly longer in [**4**]^−^ compared to **4**. Finally, the HOMO–1 for [**4**]^−^ is doubly-occupied and localized mostly on the PDI-π* system (61%).

## 3. Discussion

This work illustrates a new approach to the synthesis of bimetallic complexes featuring an Mo_2_S_2_ core. Of note, protonation of **4** to give **5** was somewhat unexpected and warrants some comment. We had hypothesized that, given two-electron oxidation of **4** affords the isolable [**4**-OTf]^+^, reaction of **4** with [Ph_2_NH_2_][BF_4_] would similarly give [**4**-BF_4_]^+^ via sequential two-electron steps; i.e., protonation of the Mo–*H* atom followed by protonation of the Cp-bound Mo. In contrast, H_2_ evolution from **4** to give **5** proceeds through, formally, one 2-electron and one single-electron step. In an effort to understand these processes, single equivalents of different anilinium acids were added to **4**. Although these reactions invariably gave mixtures, ^1^H-NMR analysis revealed that the major product was diamagnetic and still contained a MoH moiety. Importantly, a quartet at δ ~5.5 (*J* = 7 Hz) of integration 1 relative to the Mo*H* signal was consistently observed. We attribute this signal to the methine proton of a protonated imino carbon atom, with the hyperfine pattern due to coupling with the adjacent CH_3_ group. Addition of further protons to these mixtures gave **5** without discernable intermediate(s). We suggest the PDI-bound Mo in **4** is first protonated and that the resulting hydrido species rapidly transfers hydrogen to the imino-carbon[39,47]. How this complex is then protonated to give **5** is not clear, although bimolecular, homolytic H_2_ evolution from a MoH species seems likely. It is probable that similar ligand-protonation pathways give rise to the partially hydrogenated PDI observed in the cubane **6**.

**Figure 5:**
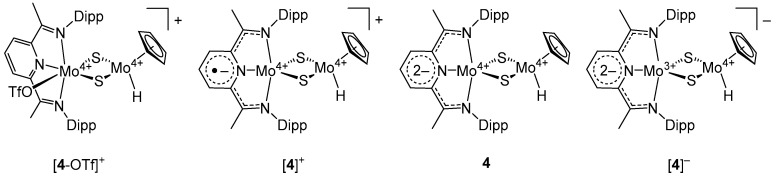
Assigned Lewis structures for the molybdenum-hydride complexes presented in this work.

Taken together, the XRD and computational data presented herein permit formulation of Lewis structure representations for the four hydride complexes. These are shown in Figure 5. In summary, on moving from [**4**-OTf]^+^ to [**4**]^+^ to **4** the PDI ligand is formally reduced in one-electron steps from PDI^0^, to PDI^●–^ to the dianion PDI^2−^. Further reduction to give [**4**]^−^ places an electron in an MO of mostly Mo(d)-character with the PDI remaining as PDI^2−^.

## 4. Conclusions

A series of site-differentiated hydrido complexes featuring an Mo_2_S_2_ core were prepared. These molecules represent rare examples of Mo–H complexes bound by sulfido ligands and are related to species proposed as intermediates in the HER catalyzed by MoS_x_ materials. Reactivity, XRD and computational studies showed that redox and protonation events for these complexes were mostly PDI-centered. This work further highlights the utility of PDI ligands as proton and electron-reservoirs. We are currently examining this class of complexes as electrocatalysts for proton reduction.

## 5. Materials and Methods

### 5.1. Synthesis

#### 5.1.1. General Considerations

Unless stated otherwise, all compounds were purchased from commercial sources and used without further purification. Solvents were dried and deoxygenated by argon sparge followed by passage through an activated alumina column and were stored over 4 Å molecular sieves. DBU was dried over CaH_2_ and distilled in vacuo. All Mo complexes, with the exception of (PDI)MoCl_3_, which could be handled in air for brief periods, were extremely air and moisture sensitive in solution and the solid state. As such, all manipulations were performed under an N_2_ atmosphere either in a glovebox or using standard Schlenk techniques. [MoCl_3_(THF)_3_] [48], (PDI)MoCl_3_ [39], PDI [49], K(C_10_H_8_)(THF)_0.5_ [50], Cp_2_Mo(SH)_2_ [40], [Ph_3_C][BAr^F^_4_] [51], [Fc][BAr^F^_4_] [52] and [Fc][OTf] [53] were synthesized according to literature procedures. [Ph_2_NH_2_][BF_4_] was prepared by addition of aqueous H[BF_4_] to a solution of Ph_2_NH in hexane and collecting the precipitate by filtration. NMR spectra were recorded at 298 K using Varian 300 MHz, 400 or 500 MHz instruments. Chemical shifts were reported in ppm relative to tetramethylsilane using residual solvent as an internal standard. IR spectra were recorded using a PerkinElmer Spectrum One FT-IR spectrometer (Waltham, MA, USA) at 4 cm^−1^ resolution. EPR X-band spectra were recorded using a Bruker EMX spectrometer (Billerica, MA, USA) and analysed using Bruker Win-EPR software. Mass spectra were recorded using either an Agilent LCTOF mass spectrometer or a Waters GCT high resolution mass spectrometer operating in LIFDI mode. CV experiments were performed using a Pine AFP1 potentiostat. The cell consisted of a glassy carbon working electrode, a Pt wire auxiliary electrode and a Pt wire pseudo-reference electrode. All potentials were referenced vs. the Fc^0/+^ couple measured as an internal standard. Elemental analyses were performed by Midwest Microlab. Solution-phase effective magnetic moments were determined by the method described by Evans [54] and corrected for diamagnetic contributions [55].

#### 5.1.2. Crystallography

X-ray intensity data were collected on a Bruker APEX2 CCD detector (Billerica, MA, USA) employing graphite-monochromated Mo-Kα radiation (λ = 0.71073 Å) at 100(1) K. Absorption and other corrections were applied using SADABS [56]. The structures were solved by direct methods using SHELXT [57] and refined against F^2^ on all data by full-matrix least squares with SHELXL-2015 [58]. Nonhydrogen atoms were refined anisotropically. Hydrogen atoms bound to Mo were permitted to refine freely; all others were refined using a riding model. CCDC 2006498–2006504 contains the supplementary crystallographic data for this paper. These data can be obtained free of charge via http://www.ccdc.cam.ac.uk/conts/retrieving.html (or from the CCDC, 12 Union Road, Cambridge CB2 1EZ, UK; Fax: +44-1223-336033; E-mail: deposit@ccdc.cam.ac.uk).

#### 5.1.3. Experimental

##### *trans*-(PDI)MoCl_2_(THF) (**1**)

(PDI)MoCl_3_ (2.04 g, 2.98 mmol) and K(C_10_H_8_)(THF)_0.5_ (0.667 g, 3.28 mmol) were suspended in toluene (20 mL). After stirring for 3 h at RT the emerald green mixture was filtered through a pad of Celite. Dilution with 3 volumes of hexane and cooling at −35 °C overnight produced pale green crystals which were collected by filtration. Recrystallisation by dissolving the complex in a minimum amount of THF and diluting with 3 volumes of hexane gave the product as glistening black-green crystals. Yield: 1.21 g, 56%. Layering a saturated THF solution of the complex with hexane gave crystals suitable for XRD studies. ^1^H NMR (C_6_D_6_, 500 MHz) δ (br) 109.60, 77.50, 32.10, 8.93, 5.07, 4.34, 2.82, 1.75, −8.42, −67.51. Evans Method (C_6_D_6_): μB 2.7 ± 0.2.

##### *trans*-(PDI)MoCl_2_(μ-SH)Mo(SH)Cp_2_ (**2**)

**1** (1.504 g, 2.09 mmol) and Cp_2_Mo(SH)_2_ (579 mg, 1.98 mmol) were suspended in THF (10 mL). After stirring at RT for 30 min a purple microcrystalline solid separated from the mixture. Toluene (20 mL) was added to the suspension and the mixture cooled to −35 °C overnight to complete precipitation. The red-purple microcrystalline solid was collected by filtration and washed with toluene (2 × 5 mL), twice with Et_2_O (2 × 5 mL) and dried under reduced pressure. Although not analytically pure, this material could be used in the next step without further purification. Yield: 1.640 g, ~88%. **2** was soluble only in chlorinated solvents in which it decomposed over several hours. Nevertheless, layering a CH_2_Cl_2_ solution of the complex with hexane at −35 °C gave crystals of sufficient quality to assign bond connectivity by XRD studies. FT-IR: cm^−1^ 1875 (SH). 

##### (PDI)Mo(μ-S)_2_Mo(η^5^-Cp)(η^1^-Cp)] (**3**)

**2** (950 mg, 1.01 mmol) was suspended in toluene (10 mL). A solution of DBU (314 mg, 2.07 mmol) in toluene (5 mL) was added dropwise with rapid stirring. After stirring at RT for 30 min the dark-red mixture was diluted with an equal volume of hexane and filtered through a pad of celite. The solvent was removed under reduced pressure and the residue recrystallized by dissolving the solid in a minimum of THF, diluting with several volumes of MeCN and concentrating to ~50% of the original volume. The resulting black-red microcrystals were collected, washed with MeCN (3 × 5 mL) and dried under reduced pressure. Peaks for impurities were observed by ^1^H NMR spectroscopy; however, this complex was sufficiently pure for the next step. Yield: 701 mg, ~80%. Layering a saturated THF solution of the complex with hexane gave crystals suitable for XRD studies. ^1^H NMR (C_6_D_6_, 400 MHz) δ 7.70 (br, 1H, pyH), 7.37 (br, 1H, pyH), 6.87 (t, *J* = 7.6 Hz, 1H, pyH), 6.18 (br, 2H, 2 × DippH), 6.05 (d, *J* = 7.7 Hz, 4H, 4 × DippH), 4.18–4.97 (m, br, 10H, 10 × CpH), 2.42 (br, 2H, CH(Me)_2_), 2.18 (br, 2H, CH(Me)_2_), 1.93 (br, 6H, 2 × CH_3_C=N), 0.99 (d, *J* = 6.7 Hz, br, 12H, 4 × CH_3_), 0.48 (d, *J* = 6.7 Hz, br, 12H, 4 × CH_3_). 

##### (PDI)Mo(μ-S)_2_Mo(η^5^-Cp)(H) (**4**)

To a mixture of **3** (204 mg, 0.235 mmol) and Na[BH_4_] (100 mg, 2.65 mmol) was added THF (5 mL) and the resulting mixture stirred at RT for 15 min to give a yellow-brown suspension. Prolonged reaction time gave decreased yields. The mixture was diluted with 2 volumes of hexane and filtered through a pad of Celite to remove excess Na[BH_4_]. The solvent was removed under reduced pressure and the crude solid suspended in Et_2_O (5 mL). A stir bar was added and the mixture stirred for 15 min before the black microcrystalline solid was collected by filtration and washed with Et_2_O (3 × 5 mL). Yield: 113 mg, 60%. **4**-D was made analogously using Na[BD_4_] (95% D). Recrystallization from THF-MeCN gave an analytical sample. Layering a saturated toluene solution of the complex with hexane gave crystals suitable for XRD studies. ^1^H-NMR (C_6_D_6_, 400 MHz) δ 7.71 (d, *J* = 7.5 Hz, 1H, 3/5-pyH), 7.58 (d, *J* = 7.5 Hz, 1H, 3/5-pyH), 6.84 (t, *J* = 7.5 Hz, 1H, 4-pyH), 6.58 (t, *J* = 7.7 Hz, 1H, 4-DippH), 6.49 (t, *J* = 7.7 Hz, 1H, 4-DippH), 6.37 (d, *J* = 7.7 Hz, 2H, 2 × 3,5-DippH), 6.28 (d, *J* = 7.7 Hz, 2H, 2 × 3/5-DippH), 5.54 (s, 5H, 5 × CpH), 2.40 (sept, *J* = 6.7 Hz, 2H, 4 × CH(Me)_2_), 2.07 (s, 3H, CH_3_C=N), 1.94 (s, 3H, CH_3_C=N), 1.15 (d, *J* = 6.6 Hz, 6H, 2 × CH_3_), 0.88 (d, *J* = 6.6 Hz, 6H, 2 × CH_3_), 0.65 (d, *J* = 6.6 Hz, 6H, 2 × CH_3_), 0.62 (d, *J* = 6.6 Hz, 6H, 2 × CH_3_), –12.58 (s, 1H, Mo–H). ^13^C {^1^H} NMR (C_6_D_6_, 101 MHz) δ 157.69, 153.85, 150.27, 147.79, 141.27, 140.10, 140.01, 139.56, 127.09, 126.72, 125.52, 124.60, 123.29, 123.09, 118.60 (13 × ArC + 2 × CH_3_C=N), 93.11 (CpC), 27.40, 27.31 (2 × CH(Me)_2_), 25.28, 25.09, 23.52, 23.49, 17.60, 17.08 (6 × CH_3_). FT-IR: cm^−1^ 3058, 2958, 2948, 2864, 1433, 1382, 1264, 1121, 959, 827, 759, 698, 637, 502. HR LIFDI MS(+): *m*/*z* 807.1475; calc. for [**4**]^+^: 807.1471. Anal. calc. for C_38_H_49_Mo_2_N_3_S_2_: C: 56.78; H: 6.14; N: 5.23. Found: C: 57.03; H: 6.35; N: 5.31.

##### [(PDI)Mo(μ-S)_2_Mo(η^5^-Cp)(H)][Na(crypt-222)] ([**4**][Na(crypt-222)])

**4** (20.1 mg, 0.0250 mmol) and freshly cut sodium (ca. 20 mg) were suspended in ether (5 mL). The mixture was stirred until no more solid **4** was visible (~1 h). The dark orange-brown solution was filtered through a pad of Celite and a solution of 2.2.2-Cryptand (10.3 mg, 0.0274 mmol) in ether (0.5 mL) added. The resulting brown precipitate was collected by filtration. Recrystallization by dissolving this material in the minimum of THF and diluting with 3 volumes of ether gave the product as brown needles. Yield: 25 mg, 84%. Layering a saturated DMAc solution of the complex with toluene gave crystals suitable for XRD studies. ^1^H-NMR (d_7_-DMF, 400 MHz) δ (br) 14.32, 13.25, 3.67 (cryptH), 3.63 (cryptH), 2.68 (cryptH), 2.11, 1.82, 1.26, −6.21. Evans Method (d_7_-DMF): μB 1.9. FT-IR: cm^−1^ 3050, 2954, 2862, 1535, 1460, 1455, 1376, 1356, 1303, 1256, 1133, 1104, 955, 755. Anal. calc. for C_56_H_85_Mo_2_N_5_NaO_6_S_2_: C: 55.89; H: 7.12; N: 5.82. Found: C: 56.05; H: 7.06; N: 5.99.

##### [(PDI)Mo(μ-S)_2_Mo(η^5^-Cp)(H)][BAr^F^_4_] ([**4**][BAr^F^_4_])

A suspension of [Ph_3_C][BAr^F^_4_] (45.0 mg, 0.0407 mmol) in toluene (2 mL) was added slowly to a stirred solution of **4** (31.1 mg, 0.0389 mmol) in toluene (2 mL). After stirring for several hours a dark blue microcrystalline solid precipitated which was collected by filtration and washed with toluene (3 × 5 mL). The crude solid was recrystallized by dissolving in the minimum of Et_2_O and layering with 3 volumes of hexane to yield large blue prisms. Crystals suitable for XRD studies were obtained similarly. Yield: 45.4 mg, 70%. ^1^H-NMR (d_8_-THF, 500 MHz) δ (br) 12.23, 7.79 (BAr^F^_4_H), 7.57 (BAr^F^_4_H), 3.7, 3.5, 3.2. Evans Method (d_8_-THF): μB 2.0 ± 0.2. FT-IR: cm^−1^ 2970, 2934, 2875, 1611, 1646, 1386, 1356, 1278, 1164, 1129 (CF_3_), 980, 886, 839, 780, 715, 682, 670. HR LIFDI MS(+): *m*/*z* 807.1477; calc. for [**4**]^+^: 807.1471. Anal. calc. for C_70_H_61_BF_24_Mo_2_N_3_S_2_.C_6_H_14_: C: 52.06; H: 4.31; N: 2.40. Found: C: 51.98; H: 4.35; N: 2.24.

##### [(PDI)Mo(OTf)(μ-S)_2_Mo(η^5^-Cp)(H)][OTf] ([4-OTf][OTf])

A suspension of [Fc][OTf] (18.7 mg, 0.0558 mmol) in THF (1 mL with an additional 2 mL for washing) was added slowly to a rapidly stirred solution of **4** (22.4 mg, 0.0279 mmol) in THF (1 mL). The solution gradually changed from yellow-brown to blue then to orange-brown. The solution was filtered through Celite, concentrated to ~0.5 mL and stored at −35 °C overnight, to produce shining red-black crystals of the complex. The supernatant was removed using a pipette and the solids washed with Et_2_O (3 × 5 mL). Yield: (25.8 mg, 84%). Recrystallization from acetone-ether at −35 °C gave crystals suitable for XRD studies. ^1^H NMR (d_6_-acetone, 400 MHz) δ 8.99 (t, *J* = 8.0 Hz, 1H, 4-pyH), 8.85 (d, *J* = 8.0 Hz, 1H, 3/5-pyH), 8.80 (d, *J* = 8.0 Hz, 1H, 3,5-pyH), 6.87 (t, *J* = 7.7 Hz, 1H, 4-DippH), 6.83 (t, *J* = 7.7 Hz, 1H, 4-DippH), 6.67 (m, br, 2H, 2 × 3/5-DippH), 6.61 (m, br, 1H, 3/5-DippH), 6.50 (m, br, 1H, 3/5-DippH), 6.19 (s, 5H, 5xCpH), 4.84 (m, br, 2H, 2 × CH(Me)_2_), 3.28 (m, br, 2H, 2 × CH(Me)_2_), 2.48 (s, 3H, CH_3_C=N), 2.38 (s, 3H, CH_3_C=N), 1.18 (m, br, 9H, 3 × CH_3_), 0.83 (m, br, 6H, 2 × CH_3_), 0.77 (m, br, 9H, 3 × CH_3_), −12.02 (s, br, 1H, Mo–H). ^19^F{^1^H} NMR (d_6_-acetone, 376 MHz) δ − 78.85 (br, O_3_SCF_3_). FT-IR: cm^−1^ 3089, 2970, 2930, 1872, 1435, 1386, 1319, 1280, 1276, 1231, 1207, 1180, 1156, 1031, 1019, 637, 519. HR LIFDI MS(+): *m*/*z* 956.1039; calc. for [**4**-OTf]+: 956.0991. Anal. calc. for C_40_H_49_F_6_Mo_2_N_3_O_6_S_4_: C: 43.60; H: 4.48; N: 3.81. Found: C: 43.45; H: 4.50; N: 3.99. 

##### [(PDI)Mo(MeCN)(μ-S)_2_Mo(η^5-^Cp)(MeCN)][BF_4_]_2_ (**5**)

**4** (30.5 mg, 0.0379 mmol) was suspended in MeCN (1 mL) and a solution of [Ph_2_NH_2_][BF_4_] (100 mg, 0.389 mmol) in MeCN (1 mL) added in one portion. The suspension rapidly cleared and vigorous gas evolution was observed. The dark purple solution was concentrated to near dryness and THF (3 mL) added. Storage at −35 °C overnight gave dark black-purple blocks which were collected by filtration, washed with THF (1 mL) and then Et_2_O (3 × 5 mL). Yield 36.1 mg, 90%. ^1^H-NMR (d_3_-MeCN, 400 MHz) δ (br) 25.90, 15.44, 15.04, 13.32, 11.19, 9.86, 9.51, 7.47, 6.11, 2.61, 2.50, 1.69, 1.05, 0.08, −1.53. Evans Method (d_3_-MeCN): μB 2.0 ± 0.1. FT-IR: cm^−1^ 3113, 2970, 2931, 1870, 2276 (CN), 2319 (CN), 1465, 1433, 1386, 1276, 1058 (BF), 815, 781, 741, 521. Anal. calc. for C_42_H_54_B_2_F_8_Mo_2_N_5_S_2_: C: 47.66; H: 5.14; N: 6.62. Found: C: 47.89; H: 5.25; N: 6.73.

##### Procedure for the Protonation of [4][Na(crypt-222)] to Give **6**:

To a solution of [**4**][Na(crypt-222)] (10.0 mg, 0.0083 mmol) in MeCN (0.5 mL) was added a solution of [Ph_2_NH_2_][BF_4_] (11 mg, 0.0043 mmol) in MeCN (0.5 mL) which caused an immediate color change from brown-orange to pale orange. Addition of Et_2_O (~3 mL) caused immediate precipitation of a small amount of amorphous brown solid which was removed by filtration through a pad of Celite. Standing for several days at RT yielded a small amount of orange-red crystals surrounded by some amorphous brown solid. The supernatant was carefully decanted using a pipette and the crystals redissolved in MeCN (~0.2 mL). Layering this solution with Et_2_O gave small crystals of **6** suitable for XRD studies (see Figure S1E).

### 5.2. DFT Calculations

All calculations were carried out using version 4.1.2 of the ORCA program package [59] Coordinates for all non-H atoms were taken from the structures determined by X-ray crystallography. To improve the efficiency of the calculations, the diisopropylphenyl and methyl groups of the PDI ligand were replaced by protons. All non-H atoms were fixed at their crystallographically-determined positions and the positions of all H atoms refined. Optimizations were performed using the Becke-Perdew GGA BP86 functional [60,61] utilizing the Ahlrichs def2-TZVP [62,63,64] basis set for all atoms. Mo atoms were treated with a 28-electron small core pseudo-potential to account for relativistic effects [65]. The contracted auxiliary Coulomb fitting basis def2/J basis was applied to all atoms [66]. Grimme’s atom-pairwise correction with Becke-Johnson damping (D3BJ) was included to account for the effects of dispersion[67]. No broken-symmetry solutions could be found for any of the molecules studied.

## Figures and Tables

**Table 1:** Selected structural metrics for **4** and its oxidized and reduced congeners.

	(Cp)Mo–S	(PDI)Mo–S	Mo–Mo	N_im_Mo	N_py_Mo	N_im_ = C_im_	C_im_–C_py_	∠MoSMo
[**4**-OTf]^+^	2.261(2)	2.2598(5)	2.770(1)	2.172(1)	2.173(1)	1.297(2)	1.473(2)	75.59(4)
	2.269(2)	2.2967(6)		2.173(1)		1.298(2)	1.467(2)	74.71(4)
[**4**]^+^	2.2790(4)	2.2433(5)	2.7832(4)	2.128(2)	2.126(1)	1.318(2)	1.440(3)	75.97(2)
	2.2332(6)	2.2921(5)		2.093(2)		1.345(2)	1.441(2)	75.90(2)
**4**	2.2419(9)	2.2912(7)	2.8237(6)	2.103(2)	2.072(2)	1.352(3)	1.406(3)	77.05(2)
	2.2964(7)	2.2525(9)		2.111(2)		1.344(2)	1.412(3)	76.72(2)
[**4**]^−^	2.291(1)	2.299(1)	2.8255(7)	2.132(2)	2.028(2)	1.364(3)	1.407(3)	75.99(4)
	2.271(1)	2.307(1)		2.162(2)		1.355(3)	1.403(3)	76.22(3)

## Supplementary Materials

The structures of **1**, **2**, **3**, **5** and **6**, selected spectroscopic data and lists of computed coordinates are available online.
