# Supplementary material for: Assembly and Redox-Rich Hydride Chemistry of an Asymmetric Mo2S2 Platform
Source: Molecules. 2020 Jul 7;25(13):3090. doi: 10.3390/molecules25133090 (PMC7411697; doi:10.3390/molecules25133090)

checkCIF/PLATON report

Structure factors have been supplied for datablock(s) hh50AM12_0m

THIS REPORT IS FOR GUIDANCE ONLY. IF USED AS PART OF A REVIEW PROCEDURE FOR PUBLICATION, IT SHOULD NOT REPLACE THE EXPERTISE OF AN EXPERIENCED CRYSTALLOGRAPHIC REFEREE.

No syntax errors found. CIF dictionary Interpreting this report

Datablock: hh50AM12_0m

```
Bond precision:  C-C = 0.0016 A          Wavelength=0.71073

Cell:           a=10.5861(3)      b=12.9206(4)     c=14.6139(5)
                alpha=71.1776(5)  beta=79.6221(5)  gamma=70.7566(5)
Temperature:    100 K

                  Calculated                  Reported
Volume            1780.17(10)                 1780.17(10)
Space group       P -1                        P -1
Hall group        -P 1                        -P 1
Moiety formula    C37 H51 Cl2 Mo N3 O         ?
Sum formula       C37 H51 Cl2 Mo N3 O         C37 H51 Cl2 Mo N3 O
Mr                720.65                      720.64
Dx,g cm-3         1.344                       1.344
Z                 2                           2
Mu (mm-1)         0.551                       0.551
F000              756.0                       756.0
F000'             753.35
h,k,lmax          14,17,20                    14,17,20
Nref              9984                        9968
Tmin,Tmax         0.756,0.914                 0.681,0.746
Tmin'             0.741

Correction method= MULTI-SCAN

Data completeness= 0.998          Theta(max)= 29.572

R(reflections)= 0.0189( 9518)     wR2(reflections)= 0.0504( 9968)

S = 1.046                  Npar= 426
```

The following ALERTS were generated. Each ALERT has the format
test-name_ALERT_alert-type_alert-level.
Click on the hyperlinks for more details of the test.

Alert level C

```
PLAT213_ALERT_2_C Atom C3D              has ADP max/min Ratio .....        3.1 prolat
PLAT732_ALERT_1_C Angle   Calc   167.43(2), Rep   167.43(1) ......       2.22 su-Rat
              CL1  -MO1  -CL2     1.555   1.555   1.555      #        15
PLAT911_ALERT_3_C Missing # FCF Refl Between THmin & STh/L=  0.600          4 Report
```

Alert level G

```
PLAT002_ALERT_2_G Number of Distance or Angle Restraints on AtSite          6 Note
PLAT003_ALERT_2_G Number of Uiso or Uij Restrained non-H Atoms ...          4 Report
PLAT176_ALERT_4_G The CIF-Embedded .res File Contains SADI Records          3 Report
PLAT177_ALERT_4_G The CIF-Embedded .res File Contains DELU Records          1 Report
PLAT232_ALERT_2_G Hirshfeld Test Diff (M-X)  Mo1    --  Cl1     ..       12.9 su
PLAT232_ALERT_2_G Hirshfeld Test Diff (M-X)  Mo1    --  Cl2     ..       12.4 su
PLAT232_ALERT_2_G Hirshfeld Test Diff (M-X)  Mo1    --  N2      ..        7.0 su
PLAT301_ALERT_3_G Main Residue  Disorder ........... Percentage =           5 Note
PLAT380_ALERT_4_G Incorrectly? Oriented X(sp2)-Methyl Moiety .....         C8 Check
PLAT860_ALERT_3_G Number of Least-Squares Restraints ............           5 Note
PLAT910_ALERT_3_G Missing # of FCF Reflections Below Th(Min) .....          2 Report
PLAT912_ALERT_4_G Missing # of FCF Reflections Above STh/L=  0.600         10 Note
```

```
   0 ALERT level A = Most likely a serious problem - resolve or explain
   0 ALERT level B = A potentially serious problem, consider carefully
   3 ALERT level C = Check. Ensure it is not caused by an omission or oversight
  12 ALERT level G = General information/check it is not something unexpected

   1 ALERT type 1 CIF construction/syntax error, inconsistent or missing data
   6 ALERT type 2 Indicator that the structure model may be wrong or deficient
   4 ALERT type 3 Indicator that the structure quality may be low
   4 ALERT type 4 Improvement, methodology, query or suggestion
   0 ALERT type 5 Informative message, check
```

It is advisable to attempt to resolve as many as possible of the alerts in all categories. Often the minor alerts point to easily fixed oversights, errors and omissions in your CIF or refinement strategy, so attention to these fine details can be worthwhile. In order to resolve some of the more serious problems it may be necessary to carry out additional measurements or structure refinements. However, the purpose of your study may justify the reported deviations and the more serious of these should normally be commented upon in the discussion or experimental section of a paper or in the "special_details" fields of the CIF. checkCIF was carefully designed to identify outliers and unusual parameters, but every test has its limitations and alerts that are not important in a particular case may appear. Conversely, the absence of alerts does not guarantee there are no aspects of the results needing attention. It is up to the individual to critically assess their own results and, if necessary, seek expert advice.

Publication of your CIF in IUCr journals

A basic structural check has been run on your CIF. These basic checks will be run on all CIFs submitted for publication in IUCr journals (*Acta Crystallographica*, *Journal of Applied Crystallography*, *Journal of Synchrotron Radiation*); however, if you intend to submit to *Acta Crystallographica Section C* or *E*, you should make sure that full publication checks are run on the final version of your CIF prior to submission.

Publication of your CIF in other journals

Please refer to the *Notes for Authors* of the relevant journal for any special instructions relating to CIF submission.

PLATON version of 20/08/2014; check.def file version of 18/08/2014

Datablock hh50AM12_0m - ellipsoid plot

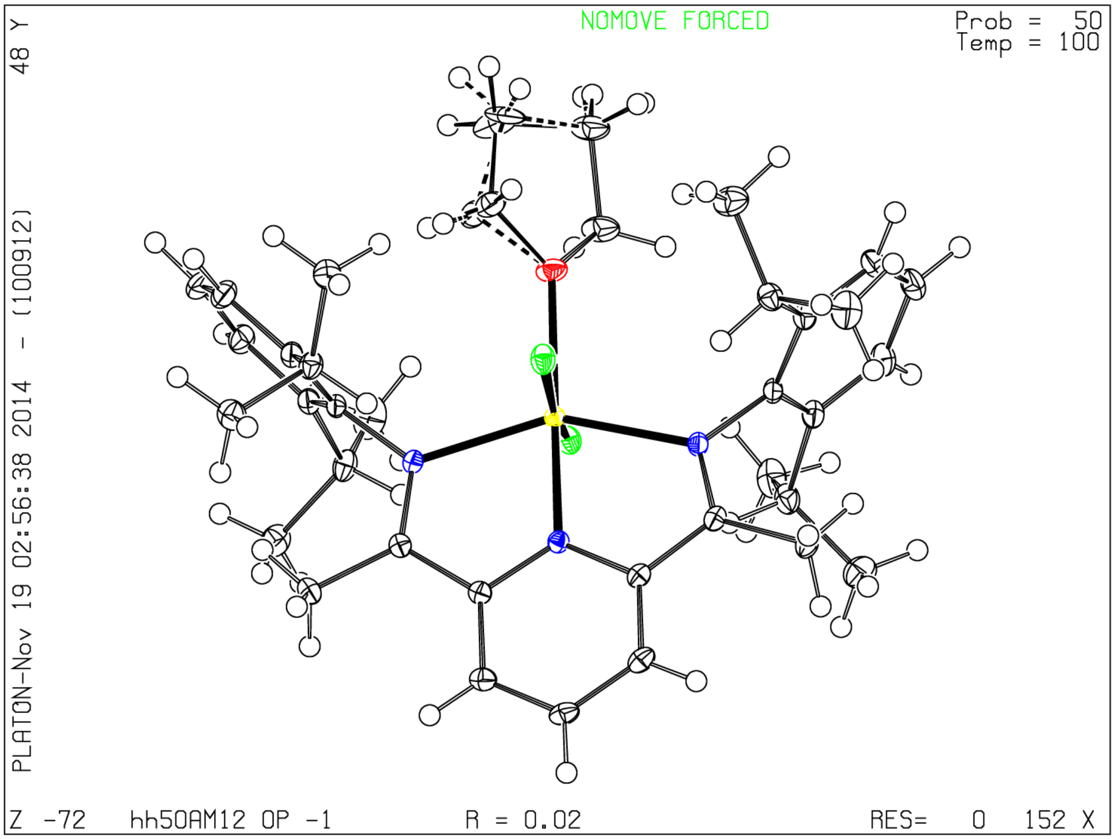

checkCIF/PLATON report

Structure factors have been supplied for datablock(s) hh85AM29_0m

THIS REPORT IS FOR GUIDANCE ONLY. IF USED AS PART OF A REVIEW PROCEDURE FOR PUBLICATION, IT SHOULD NOT REPLACE THE EXPERTISE OF AN EXPERIENCED CRYSTALLOGRAPHIC REFEREE.

No syntax errors found. CIF dictionary Interpreting this report

Datablock: hh85AM29_0m

```
Bond precision:  C-C = 0.0031 A          Wavelength=0.71073

Cell:           a=20.5549(6)    b=12.5715(4)        c=29.0368(9)
                alpha=90        beta=107.0914(5)    gamma=90
Temperature:    100 K

                Calculated                     Reported
Volume          7171.9(4)                      7171.9(4)
Space group     C 2/c                          C 2/c
Hall group      -C 2yc                         -C 2yc
Moiety formula  C38 H49 Mo2 N3 S2              ?
Sum formula     C38 H49 Mo2 N3 S2              C38 H49 Mo2 N3 S2
Mr              803.80                         803.80
Dx,g cm-3       1.489                          1.489
Z               8                              8
Mu (mm-1)       0.847                          0.847
F000            3312.0                         3312.0
F000'           3287.14
h,k,lmax        28,17,40                       28,17,40
Nref            10075                          10071
Tmin,Tmax       0.759,0.798                    0.701,0.746
Tmin'           0.692

Correction method= # Reported T Limits: Tmin=0.701 Tmax=0.746
AbsCorr = MULTI-SCAN

Data completeness= 1.000          Theta(max)= 29.574

R(reflections)= 0.0334( 9383)     wR2(reflections)= 0.0657( 10071)

S = 1.229                   Npar= 468
```

The following ALERTS were generated. Each ALERT has the format
test-name_ALERT_alert-type_alert-level.
Click on the hyperlinks for more details of the test.

Alert level C

PLAT220_ALERT_2_C	Non-Solvent Resd 1 C Ueq(max)/Ueq(min) Range	3.5 Ratio
PLAT906_ALERT_3_C	Large K value in the Analysis of Variance	4.017 Check
PLAT972_ALERT_2_C	Check Calcd Residual Density 0.74A From Mo2	-1.87 eA-3

Alert level G

PLAT002_ALERT_2_G	Number of Distance or Angle Restraints on AtSite	3 Note
PLAT003_ALERT_2_G	Number of Uiso or Uij Restrained non-H Atoms ...	10 Report
PLAT083_ALERT_2_G	SHELXL Second Parameter in WGHT Unusually Large	25.01 Why ?
PLAT176_ALERT_4_G	The CIF-Embedded .res File Contains SADI Records	1 Report
PLAT177_ALERT_4_G	The CIF-Embedded .res File Contains DELU Records	1 Report
PLAT232_ALERT_2_G	Hirshfeld Test Diff (M-X) Mo1 -- S1 ..	6.0 s.u.
PLAT301_ALERT_3_G	Main Residue Disorder .... Percentage =	11 Note
PLAT860_ALERT_3_G	Number of Least-Squares Restraints	21 Note
PLAT910_ALERT_3_G	Missing # of FCF Reflection(s) Below Theta(Min)	1 Note
PLAT912_ALERT_4_G	Missing # of FCF Reflections Above STh/L= 0.600	3 Note
PLAT978_ALERT_2_G	Number C-C Bonds with Positive Residual Density	15 Note

0 **ALERT level A** = Most likely a serious problem - resolve or explain
0 **ALERT level B** = A potentially serious problem, consider carefully
3 **ALERT level C** = Check. Ensure it is not caused by an omission or oversight
11 **ALERT level G** = General information/check it is not something unexpected

0 ALERT type 1 CIF construction/syntax error, inconsistent or missing data
7 ALERT type 2 Indicator that the structure model may be wrong or deficient
4 ALERT type 3 Indicator that the structure quality may be low
3 ALERT type 4 Improvement, methodology, query or suggestion
0 ALERT type 5 Informative message, check

It is advisable to attempt to resolve as many as possible of the alerts in all categories. Often the minor alerts point to easily fixed oversights, errors and omissions in your CIF or refinement strategy, so attention to these fine details can be worthwhile. In order to resolve some of the more serious problems it may be necessary to carry out additional measurements or structure refinements. However, the purpose of your study may justify the reported deviations and the more serious of these should normally be commented upon in the discussion or experimental section of a paper or in the "special_details" fields of the CIF. checkCIF was carefully designed to identify outliers and unusual parameters, but every test has its limitations and alerts that are not important in a particular case may appear. Conversely, the absence of alerts does not guarantee there are no aspects of the results needing attention. It is up to the individual to critically assess their own results and, if necessary, seek expert advice.

Publication of your CIF in IUCr journals

A basic structural check has been run on your CIF. These basic checks will be run on all CIFs submitted for publication in IUCr journals (*Acta Crystallographica*, *Journal of Applied Crystallography*, *Journal of Synchrotron Radiation*); however, if you intend to submit to *Acta Crystallographica Section C* or *E* or *IUCrData*, you should make sure that full publication checks are run on the final version of your CIF prior to submission.

Publication of your CIF in other journals

Please refer to the *Notes for Authors* of the relevant journal for any special instructions relating to CIF submission.

PLATON version of 06/05/2016; check.def file version of 05/05/2016

Datablock hh85AM29_0m - ellipsoid plot

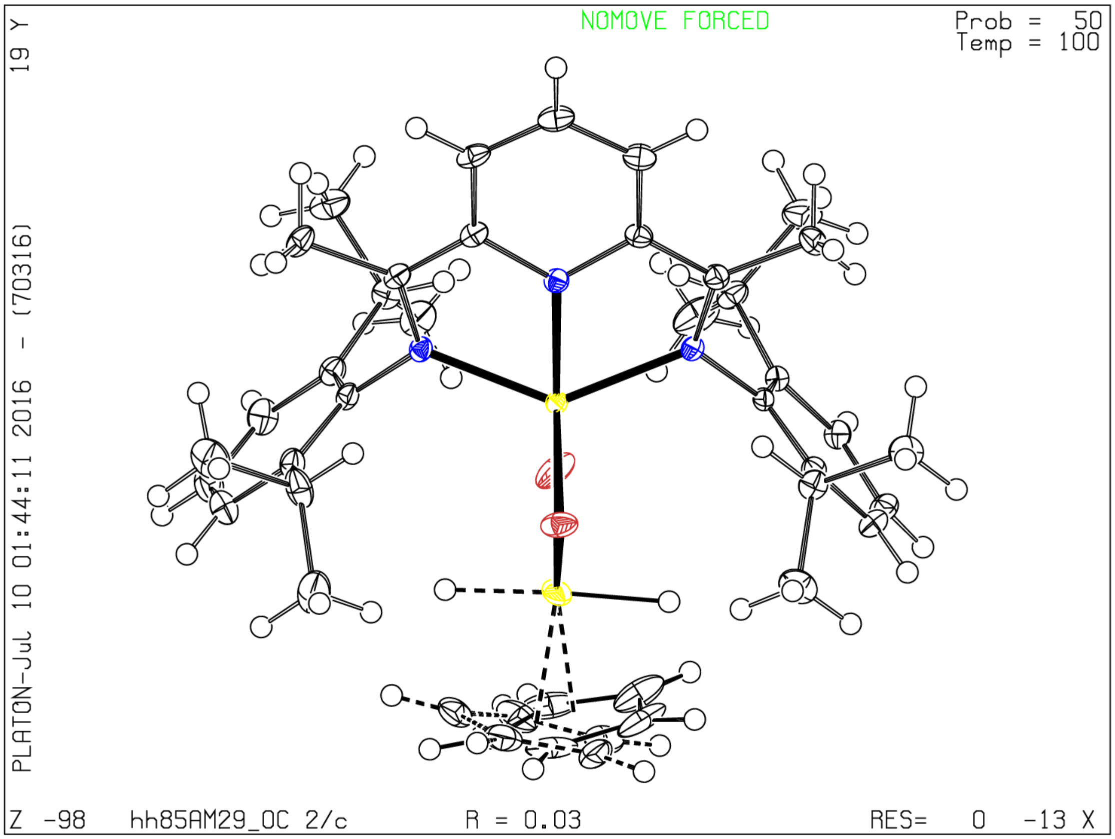

checkCIF/PLATON report

Structure factors have been supplied for datablock(s) hh52AM14r_0m-5

THIS REPORT IS FOR GUIDANCE ONLY. IF USED AS PART OF A REVIEW PROCEDURE FOR PUBLICATION, IT SHOULD NOT REPLACE THE EXPERTISE OF AN EXPERIENCED CRYSTALLOGRAPHIC REFEREE.

No syntax errors found. CIF dictionary Interpreting this report

Datablock: hh52AM14r_0m-5

```
Bond precision:  C-C = 0.0087 A         Wavelength=0.71073

Cell:         a=11.1885(11)     b=14.8249(14)     c=26.397(3)
              alpha=96.3680(15) beta=101.6756(15) gamma=110.1426(14)
Temperature: 100 K

                Calculated                 Reported
Volume          3947.2(7)                  3947.2(7)
Space group     P -1                       P -1
Hall group      -P 1                       -P 1
Moiety formula  C43 H53 Mo2 N3 S2          ?
Sum formula     C43 H53 Mo2 N3 S2          C43 H53 Mo2 N3 S2
Mr              867.88                     867.88
Dx,g cm-3       1.460                      1.460
Z               4                          4
Mu (mm-1)       0.775                      0.775
F000            1792.0                     1792.0
F000'           1779.62
h,k,lmax        13,18,32                   13,18,32
Nref            16121                      15663
Tmin,Tmax       0.857,0.981                0.813,0.928
Tmin'           0.678

Correction method= # Reported T Limits: Tmin=0.813 Tmax=0.928
AbsCorr = MULTI-SCAN

Data completeness= 0.972          Theta(max)= 26.372

R(reflections)= 0.0402( 14509)    wR2(reflections)= 0.1069( 15663)

S = 1.093                 Npar= 922
```

The following ALERTS were generated. Each ALERT has the format
test-name_ALERT_alert-type_alert-level.
Click on the hyperlinks for more details of the test.

Alert level C

```
PLAT029_ALERT_3_C _diffrn_measured_fraction_theta_full value Low .      0.974 Note
PLAT213_ALERT_2_C Atom C18A            has ADP max/min Ratio .....        3.7 oblate
PLAT342_ALERT_3_C Low Bond Precision on  C-C Bonds ..............    0.00874 Ang.
PLAT601_ALERT_2_C Structure Contains Solvent Accessible VOIDS of .         80 Ang3
PLAT911_ALERT_3_C Missing # FCF Refl Between THmin & STh/L=  0.600        853 Report
```

Alert level G

```
PLAT083_ALERT_2_G SHELXL Second Parameter in WGHT  Unusually Large      20.63 Why ?
PLAT870_ALERT_4_G ALERTS Related to Twinning Effects Suppressed ..          ! Info
PLAT910_ALERT_3_G Missing # of FCF Reflection(s) Below Theta(Min)           1 Note
PLAT912_ALERT_4_G Missing # of FCF Reflections Above STh/L=  0.600        309 Note
PLAT931_ALERT_5_G Found Twin Law (       )[ 1 0  0] Estimated BASF       0.26 Check
```

0 **ALERT level A** = Most likely a serious problem - resolve or explain
0 **ALERT level B** = A potentially serious problem, consider carefully
5 **ALERT level C** = Check. Ensure it is not caused by an omission or oversight
5 **ALERT level G** = General information/check it is not something unexpected

0 ALERT type 1 CIF construction/syntax error, inconsistent or missing data
3 ALERT type 2 Indicator that the structure model may be wrong or deficient
4 ALERT type 3 Indicator that the structure quality may be low
2 ALERT type 4 Improvement, methodology, query or suggestion
1 ALERT type 5 Informative message, check

It is advisable to attempt to resolve as many as possible of the alerts in all categories. Often the minor alerts point to easily fixed oversights, errors and omissions in your CIF or refinement strategy, so attention to these fine details can be worthwhile. In order to resolve some of the more serious problems it may be necessary to carry out additional measurements or structure refinements. However, the purpose of your study may justify the reported deviations and the more serious of these should normally be commented upon in the discussion or experimental section of a paper or in the "special_details" fields of the CIF. checkCIF was carefully designed to identify outliers and unusual parameters, but every test has its limitations and alerts that are not important in a particular case may appear. Conversely, the absence of alerts does not guarantee there are no aspects of the results needing attention. It is up to the individual to critically assess their own results and, if necessary, seek expert advice.

Publication of your CIF in IUCr journals

A basic structural check has been run on your CIF. These basic checks will be run on all CIFs submitted for publication in IUCr journals (*Acta Crystallographica*, *Journal of Applied Crystallography*, *Journal of Synchrotron Radiation*); however, if you intend to submit to *Acta Crystallographica Section C* or *E* or *IUCrData*, you should make sure that full publication checks are run on the final version of your CIF prior to submission.

Publication of your CIF in other journals

Please refer to the *Notes for Authors* of the relevant journal for any special instructions relating to CIF submission.

PLATON version of 06/05/2016; check.def file version of 05/05/2016

Datablock hh52AM14r_0m-5 - ellipsoid plot

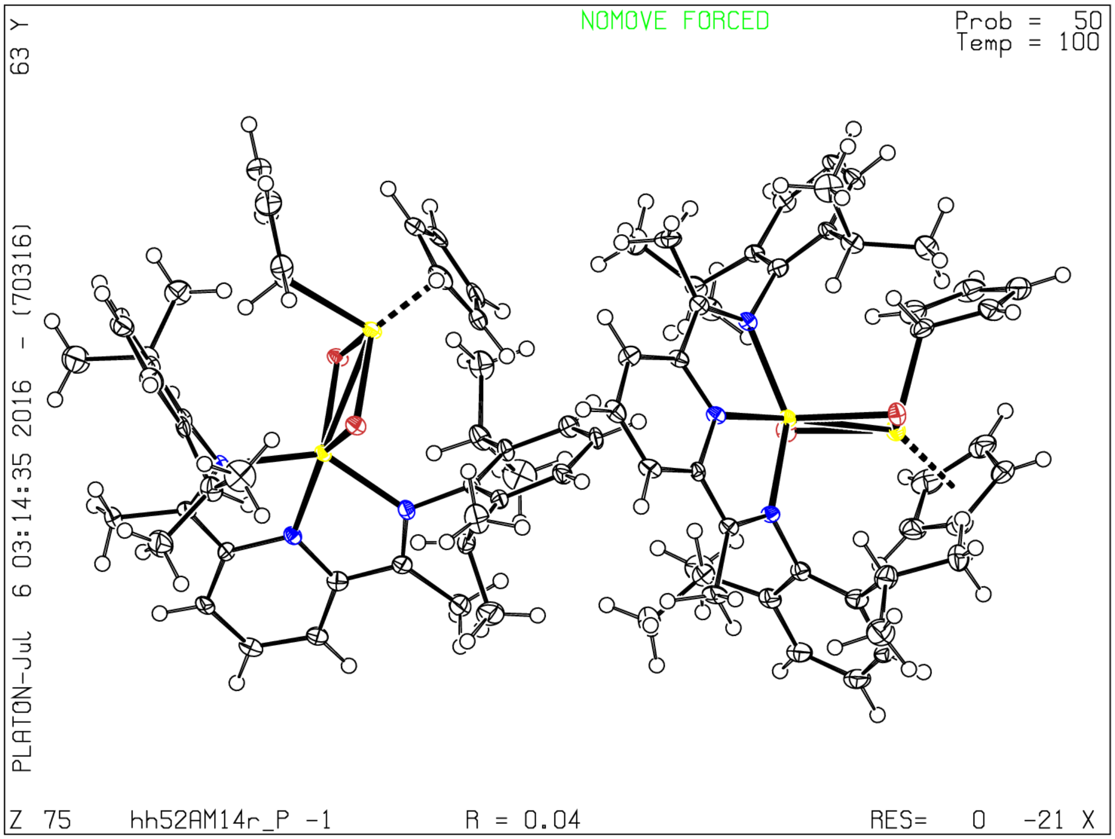

checkCIF/PLATON report

Structure factors have been supplied for datablock(s) hh148AM54r_0m

THIS REPORT IS FOR GUIDANCE ONLY. IF USED AS PART OF A REVIEW PROCEDURE FOR PUBLICATION, IT SHOULD NOT REPLACE THE EXPERTISE OF AN EXPERIENCED CRYSTALLOGRAPHIC REFEREE.

No syntax errors found. CIF dictionary Interpreting this report

Datablock: hh148AM54r_0m

```
Bond precision:  C-C = 0.0035 A          Wavelength=0.71073

Cell:            a=42.8652(17)     b=49.0867(19)     c=12.2689(5)
                 alpha=90          beta=90           gamma=90
Temperature:     100 K
```

	Calculated	Reported
Volume	25815.1(18)	25815.1(18)
Space group	F d d 2	F d d 2
Hall group	F 2 -2d	F 2 -2d
Moiety formula	4(C38 H49 Mo2 N3 S2), 4(C18 H36 N2 Na O6), C14 H16, 2(C7 H8), 2	?
Sum formula	C260 H390 Mo8 N22 Na4 O26 S8	C65 H97.50 Mo2 N5.50 Na O6.50 S2
Mr	5355.90	1338.97
Dx,g cm-3	1.378	1.378
Z	4	16
Mu (mm-1)	0.515	0.515
F000	11280.0	11280.0
F000'	11232.83	
h,k,lmax	61,70,17	61,70,17
Nref	19708[10276]	19698
Tmin,Tmax	0.908,0.961	0.881,0.928
Tmin'	0.833	

```
Correction method= # Reported T Limits: Tmin=0.881 Tmax=0.928
AbsCorr = NONE

Data completeness= 1.92/1.00      Theta(max)= 30.508

R(reflections)= 0.0243( 18438)    wR2(reflections)= 0.0569( 19698)

S = 1.065                  Npar= 875
```

The following ALERTS were generated. Each ALERT has the format
test-name_ALERT_alert-type_alert-level.
Click on the hyperlinks for more details of the test.

Alert level B

PLAT250_ALERT_2_B	Large U3/U1 Ratio for Average U(i,j) Tensor	4.8	Note

Alert level C

PLAT250_ALERT_2_C	Large U3/U1 Ratio for Average U(i,j) Tensor	3.4	Note
PLAT413_ALERT_2_C	Short Inter XH3 .. XHn H33C .. H3I ..	2.06	Ang.
PLAT910_ALERT_3_C	Missing # of FCF Reflection(s) Below Theta(Min)	6	Note

Alert level G

PLAT002_ALERT_2_G	Number of Distance or Angle Restraints on AtSite	6	Note
PLAT003_ALERT_2_G	Number of Uiso or Uij Restrained non-H Atoms ...	27	Report
PLAT045_ALERT_1_G	Calculated and Reported Z Differ by a Factor ...	0.25	Check
PLAT083_ALERT_2_G	SHELXL Second Parameter in WGHT Unusually Large	12.07	Why ?
PLAT174_ALERT_4_G	The CIF-Embedded .res File Contains FLAT Records	2	Report
PLAT176_ALERT_4_G	The CIF-Embedded .res File Contains SADI Records	2	Report
PLAT177_ALERT_4_G	The CIF-Embedded .res File Contains DELU Records	1	Report
PLAT178_ALERT_4_G	The CIF-Embedded .res File Contains SIMU Records	1	Report
PLAT232_ALERT_2_G	Hirshfeld Test Diff (M-X) Mo2 -- S2 ..	6.0	s.u.
PLAT300_ALERT_4_G	Atom Site Occupancy of <C1T is Constrained at	0.25	Check
PLAT300_ALERT_4_G	Atom Site Occupancy of <C1U is Constrained at	0.25	Check
PLAT300_ALERT_4_G	Atom Site Occupancy of <C2T is Constrained at	0.25	Check
PLAT300_ALERT_4_G	Atom Site Occupancy of <C2U is Constrained at	0.25	Check
PLAT300_ALERT_4_G	Atom Site Occupancy of <C3T is Constrained at	0.25	Check
PLAT300_ALERT_4_G	Atom Site Occupancy of <C3U is Constrained at	0.25	Check
PLAT300_ALERT_4_G	Atom Site Occupancy of <C4T is Constrained at	0.25	Check
PLAT300_ALERT_4_G	Atom Site Occupancy of <C4U is Constrained at	0.25	Check
PLAT300_ALERT_4_G	Atom Site Occupancy of <C5T is Constrained at	0.25	Check
PLAT300_ALERT_4_G	Atom Site Occupancy of <C5U is Constrained at	0.25	Check
PLAT300_ALERT_4_G	Atom Site Occupancy of <C6T is Constrained at	0.25	Check
PLAT300_ALERT_4_G	Atom Site Occupancy of <C6U is Constrained at	0.25	Check
PLAT300_ALERT_4_G	Atom Site Occupancy of <C7T is Constrained at	0.25	Check
PLAT300_ALERT_4_G	Atom Site Occupancy of <C7U is Constrained at	0.25	Check
PLAT300_ALERT_4_G	Atom Site Occupancy of <H2D is Constrained at	0.25	Check
PLAT300_ALERT_4_G	Atom Site Occupancy of <H2E is Constrained at	0.25	Check
PLAT300_ALERT_4_G	Atom Site Occupancy of <H3H is Constrained at	0.25	Check
PLAT300_ALERT_4_G	Atom Site Occupancy of <H3I is Constrained at	0.25	Check
PLAT300_ALERT_4_G	Atom Site Occupancy of <H4H is Constrained at	0.25	Check
PLAT300_ALERT_4_G	Atom Site Occupancy of <H4I is Constrained at	0.25	Check
PLAT300_ALERT_4_G	Atom Site Occupancy of <H5E is Constrained at	0.25	Check
PLAT300_ALERT_4_G	Atom Site Occupancy of <H5F is Constrained at	0.25	Check
PLAT300_ALERT_4_G	Atom Site Occupancy of <H6D is Constrained at	0.25	Check
PLAT300_ALERT_4_G	Atom Site Occupancy of <H6E is Constrained at	0.25	Check
PLAT300_ALERT_4_G	Atom Site Occupancy of <H7F is Constrained at	0.25	Check
PLAT300_ALERT_4_G	Atom Site Occupancy of <H7G is Constrained at	0.25	Check
PLAT300_ALERT_4_G	Atom Site Occupancy of <H7H is Constrained at	0.25	Check
PLAT300_ALERT_4_G	Atom Site Occupancy of <H7I is Constrained at	0.25	Check
PLAT300_ALERT_4_G	Atom Site Occupancy of <H7J is Constrained at	0.25	Check
PLAT300_ALERT_4_G	Atom Site Occupancy of <H7K is Constrained at	0.25	Check
PLAT300_ALERT_4_G	Atom Site Occupancy of *C1S is Constrained at	0.5	Check
PLAT300_ALERT_4_G	Atom Site Occupancy of *C2S is Constrained at	0.5	Check
PLAT300_ALERT_4_G	Atom Site Occupancy of *C3S is Constrained at	0.5	Check
PLAT300_ALERT_4_G	Atom Site Occupancy of *C4S is Constrained at	0.5	Check
PLAT300_ALERT_4_G	Atom Site Occupancy of *C5S is Constrained at	0.5	Check

```
PLAT300 ALERT 4 G Atom Site Occupancy of *C6S    is Constrained at        0.5 Check
PLAT300 ALERT 4 G Atom Site Occupancy of *C7S    is Constrained at        0.5 Check
PLAT300 ALERT 4 G Atom Site Occupancy of *H2C    is Constrained at        0.5 Check
PLAT300 ALERT 4 G Atom Site Occupancy of *H3G    is Constrained at        0.5 Check
PLAT300 ALERT 4 G Atom Site Occupancy of *H4G    is Constrained at        0.5 Check
PLAT300 ALERT 4 G Atom Site Occupancy of *H5D    is Constrained at        0.5 Check
PLAT300 ALERT 4 G Atom Site Occupancy of *H6C    is Constrained at        0.5 Check
PLAT300 ALERT 4 G Atom Site Occupancy of *H7C    is Constrained at        0.5 Check
PLAT300 ALERT 4 G Atom Site Occupancy of *H7D    is Constrained at        0.5 Check
PLAT300 ALERT 4 G Atom Site Occupancy of *H7E    is Constrained at        0.5 Check
PLAT300 ALERT 4 G Atom Site Occupancy of *O1D    is Constrained at        0.5 Check
PLAT300 ALERT 4 G Atom Site Occupancy of *N1D    is Constrained at        0.5 Check
PLAT300 ALERT 4 G Atom Site Occupancy of *C1D    is Constrained at        0.5 Check
PLAT300 ALERT 4 G Atom Site Occupancy of *C2D    is Constrained at        0.5 Check
PLAT300 ALERT 4 G Atom Site Occupancy of *C3D    is Constrained at        0.5 Check
PLAT300 ALERT 4 G Atom Site Occupancy of *C4D    is Constrained at        0.5 Check
PLAT300 ALERT 4 G Atom Site Occupancy of *H1C    is Constrained at        0.5 Check
PLAT300 ALERT 4 G Atom Site Occupancy of *H1D    is Constrained at        0.5 Check
PLAT300 ALERT 4 G Atom Site Occupancy of *H1E    is Constrained at        0.5 Check
PLAT300 ALERT 4 G Atom Site Occupancy of *H3D    is Constrained at        0.5 Check
PLAT300 ALERT 4 G Atom Site Occupancy of *H3E    is Constrained at        0.5 Check
PLAT300 ALERT 4 G Atom Site Occupancy of *H3F    is Constrained at        0.5 Check
PLAT300 ALERT 4 G Atom Site Occupancy of *H4D    is Constrained at        0.5 Check
PLAT300 ALERT 4 G Atom Site Occupancy of *H4E    is Constrained at        0.5 Check
PLAT300 ALERT 4 G Atom Site Occupancy of *H4F    is Constrained at        0.5 Check
PLAT301 ALERT 3 G Main Residue  Disorder ............ Percentage =          5 Note
PLAT302 ALERT 4 G Anion/Solvent Disorder ............ Percentage =        100 Note
PLAT304 ALERT 4 G Non-Integer Number of Atoms (   7.50) in Resd. #          3 Check
PLAT304 ALERT 4 G Non-Integer Number of Atoms (   7.50) in Resd. #          4 Check
PLAT304 ALERT 4 G Non-Integer Number of Atoms (   7.50) in Resd. #          5 Check
PLAT789 ALERT 4 G Atoms with Negative _atom_site_disorder_group  #         30 Check
PLAT860 ALERT 3 G Number of Least-Squares Restraints ............         481 Note
PLAT978 ALERT 2 G Number C-C Bonds with Positive Residual Density           9 Note
```

```
   0 ALERT level A = Most likely a serious problem - resolve or explain
   1 ALERT level B = A potentially serious problem, consider carefully
   3 ALERT level C = Check. Ensure it is not caused by an omission or oversight
  77 ALERT level G = General information/check it is not something unexpected

   1 ALERT type 1 CIF construction/syntax error, inconsistent or missing data
   8 ALERT type 2 Indicator that the structure model may be wrong or deficient
   3 ALERT type 3 Indicator that the structure quality may be low
  69 ALERT type 4 Improvement, methodology, query or suggestion
   0 ALERT type 5 Informative message, check
```

It is advisable to attempt to resolve as many as possible of the alerts in all categories. Often the minor alerts point to easily fixed oversights, errors and omissions in your CIF or refinement strategy, so attention to these fine details can be worthwhile. In order to resolve some of the more serious problems it may be necessary to carry out additional measurements or structure refinements. However, the purpose of your study may justify the reported deviations and the more serious of these should normally be commented upon in the discussion or experimental section of a paper or in the "special_details" fields of the CIF. checkCIF was carefully designed to identify outliers and unusual parameters, but every test has its limitations and alerts that are not important in a particular case may appear. Conversely, the absence of alerts does not guarantee there are no aspects of the results needing attention. It is up to the individual to critically assess their own results and, if necessary, seek expert advice.

Publication of your CIF in IUCr journals

A basic structural check has been run on your CIF. These basic checks will be run on all CIFs submitted for publication in IUCr journals (*Acta Crystallographica*, *Journal of Applied Crystallography*, *Journal of Synchrotron Radiation*); however, if you intend to submit to *Acta Crystallographica Section C* or *E* or *IUCrData*, you should make sure that full publication checks are run on the final version of your CIF prior to submission.

Publication of your CIF in other journals

Please refer to the *Notes for Authors* of the relevant journal for any special instructions relating to CIF submission.

PLATON version of 06/05/2016; check.def file version of 05/05/2016

Datablock hh148AM54r_0m - ellipsoid plot

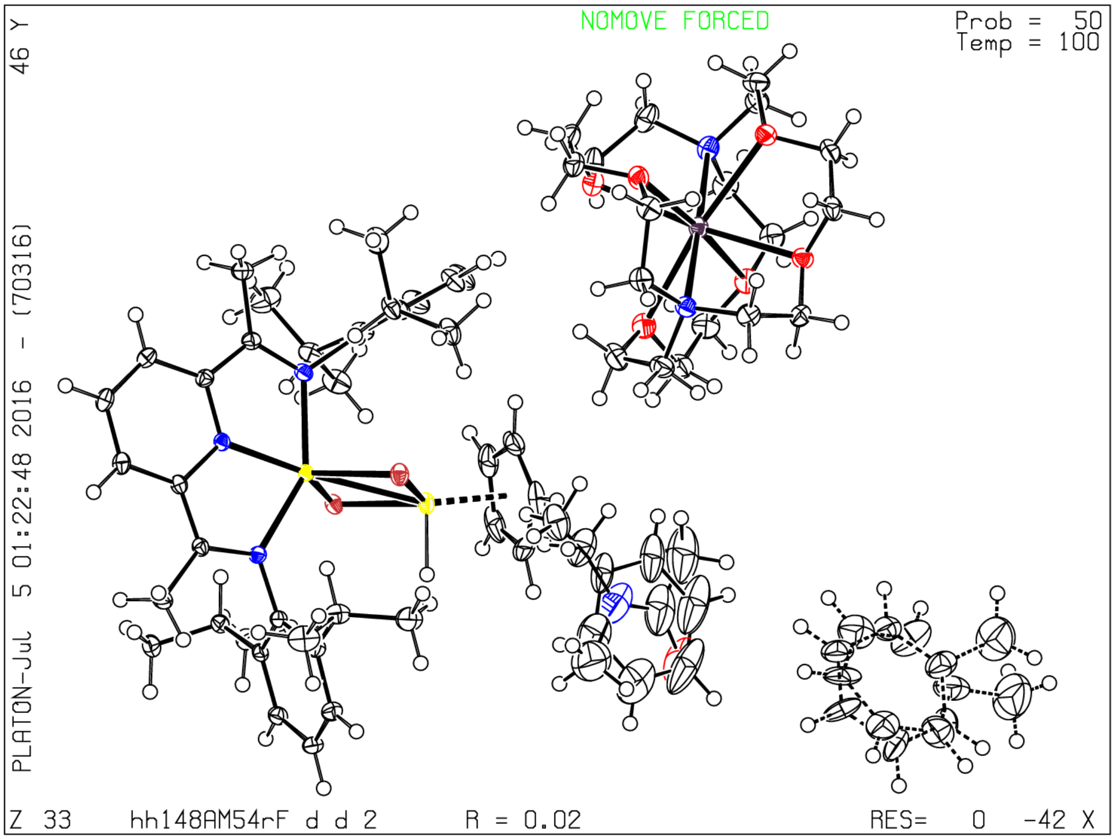

checkCIF/PLATON report

Structure factors have been supplied for datablock(s) hh172AM60_0m

THIS REPORT IS FOR GUIDANCE ONLY. IF USED AS PART OF A REVIEW PROCEDURE FOR PUBLICATION, IT SHOULD NOT REPLACE THE EXPERTISE OF AN EXPERIENCED CRYSTALLOGRAPHIC REFEREE.

No syntax errors found. CIF dictionary Interpreting this report

Datablock: hh172AM60_0m

```
Bond precision:  C-C = 0.0028 A          Wavelength=0.71073

Cell:         a=12.7387(3)       b=16.7266(4)      c=19.0589(4)
              alpha=104.0257(11) beta=107.7631(11) gamma=91.0793(12)
Temperature: 100 K
```

	Calculated	Reported
Volume	3733.11(15)	3733.11(15)
Space group	P -1	P -1
Hall group	-P 1	-P 1
Moiety formula	C32 H12 B F24, C38 H49 Mo2 N3 S2, C4 H10 O	?
Sum formula	C74 H71 B F24 Mo2 N3 O S2	C74 H71 B F24 Mo2 N3 O S2
Mr	1741.15	1741.14
Dx,g cm-3	1.549	1.549
Z	2	2
Mu (mm-1)	0.498	0.498
F000	1762.0	1762.0
F000'	1756.77	
h,k,lmax	18,23,27	18,23,27
Nref	22780	22766
Tmin,Tmax	0.810,0.848	0.805,0.862
Tmin'	0.788	

```
Correction method= # Reported T Limits: Tmin=0.805 Tmax=0.862
AbsCorr = MULTI-SCAN

Data completeness= 0.999          Theta(max)= 30.508

R(reflections)= 0.0371( 20259)    wR2(reflections)= 0.0978( 22766)

S = 1.043                   Npar= 1028
```

The following ALERTS were generated. Each ALERT has the format
test-name_ALERT_alert-type_alert-level.
Click on the hyperlinks for more details of the test.

Alert level B

```
PLAT213_ALERT_2_B Atom F11A          has ADP max/min Ratio .....        4.4 prolat
PLAT213_ALERT_2_B Atom F24A          has ADP max/min Ratio .....        4.2 prolat
```

Alert level C

```
PLAT213_ALERT_2_C Atom F4A           has ADP max/min Ratio .....        3.4 prolat
PLAT213_ALERT_2_C Atom F12A          has ADP max/min Ratio .....        3.2 prolat
PLAT220_ALERT_2_C Non-Solvent Resd 1    F   Ueq(max)/Ueq(min) Range     3.1 Ratio
PLAT412_ALERT_2_C Short Intra XH3 .. XHn      H8A     ..   H2N     ..  1.84 Ang.
PLAT910_ALERT_3_C Missing # of FCF Reflection(s) Below Theta(Min)        10 Note
PLAT911_ALERT_3_C Missing # FCF Refl Between THmin & STh/L=  0.600        5 Report
PLAT971_ALERT_2_C Check Calcd Residual Density  0.78A From    F14A     1.52 eA-3
PLAT978_ALERT_2_C Number C-C Bonds with Positive Residual Density         0 Note
```

Alert level G

```
PLAT002_ALERT_2_G Number of Distance or Angle Restraints on AtSite        8 Note
PLAT003_ALERT_2_G Number of Uiso or Uij Restrained non-H Atoms ...       10 Report
PLAT176_ALERT_4_G The CIF-Embedded .res File Contains SADI Records        2 Report
PLAT177_ALERT_4_G The CIF-Embedded .res File Contains DELU Records        1 Report
PLAT232_ALERT_2_G Hirshfeld Test Diff (M-X)  Mo1     --   N1       ..   8.2 s.u.
PLAT242_ALERT_2_G Low    'MainMol' Ueq as Compared to Neighbors of      C7A Check
PLAT242_ALERT_2_G Low    'MainMol' Ueq as Compared to Neighbors of      C8A Check
PLAT242_ALERT_2_G Low    'MainMol' Ueq as Compared to Neighbors of     C15A Check
PLAT242_ALERT_2_G Low    'MainMol' Ueq as Compared to Neighbors of     C16A Check
PLAT242_ALERT_2_G Low    'MainMol' Ueq as Compared to Neighbors of     C23A Check
PLAT242_ALERT_2_G Low    'MainMol' Ueq as Compared to Neighbors of     C24A Check
PLAT242_ALERT_2_G Low    'MainMol' Ueq as Compared to Neighbors of     C31A Check
PLAT242_ALERT_2_G Low    'MainMol' Ueq as Compared to Neighbors of     C32A Check
PLAT301_ALERT_3_G Main Residue  Disorder ............ Percentage =        5 Note
PLAT434_ALERT_2_G Short Inter HL..HL Contact F9A     ..   F9A      ..  2.81 Ang.
PLAT860_ALERT_3_G Number of Least-Squares Restraints ............        31 Note
```

---

0 **ALERT level A** = Most likely a serious problem - resolve or explain
2 **ALERT level B** = A potentially serious problem, consider carefully
8 **ALERT level C** = Check. Ensure it is not caused by an omission or oversight
16 **ALERT level G** = General information/check it is not something unexpected

0 ALERT type 1 CIF construction/syntax error, inconsistent or missing data
20 ALERT type 2 Indicator that the structure model may be wrong or deficient
4 ALERT type 3 Indicator that the structure quality may be low
2 ALERT type 4 Improvement, methodology, query or suggestion
0 ALERT type 5 Informative message, check

It is advisable to attempt to resolve as many as possible of the alerts in all categories. Often the minor alerts point to easily fixed oversights, errors and omissions in your CIF or refinement strategy, so attention to these fine details can be worthwhile. In order to resolve some of the more serious problems it may be necessary to carry out additional measurements or structure refinements. However, the purpose of your study may justify the reported deviations and the more serious of these should normally be commented upon in the discussion or experimental section of a paper or in the "special_details" fields of the CIF. checkCIF was carefully designed to identify outliers and unusual parameters, but every test has its limitations and alerts that are not important in a particular case may appear. Conversely, the absence of alerts does not guarantee there are no aspects of the results needing attention. It is up to the individual to critically assess their own results and, if necessary, seek expert advice.

Publication of your CIF in IUCr journals

A basic structural check has been run on your CIF. These basic checks will be run on all CIFs submitted for publication in IUCr journals (*Acta Crystallographica*, *Journal of Applied Crystallography*, *Journal of Synchrotron Radiation*); however, if you intend to submit to *Acta Crystallographica Section C* or *E* or *IUCrData*, you should make sure that full publication checks are run on the final version of your CIF prior to submission.

Publication of your CIF in other journals

Please refer to the *Notes for Authors* of the relevant journal for any special instructions relating to CIF submission.

PLATON version of 06/05/2016; check.def file version of 05/05/2016

Datablock hh172AM60_0m - ellipsoid plot

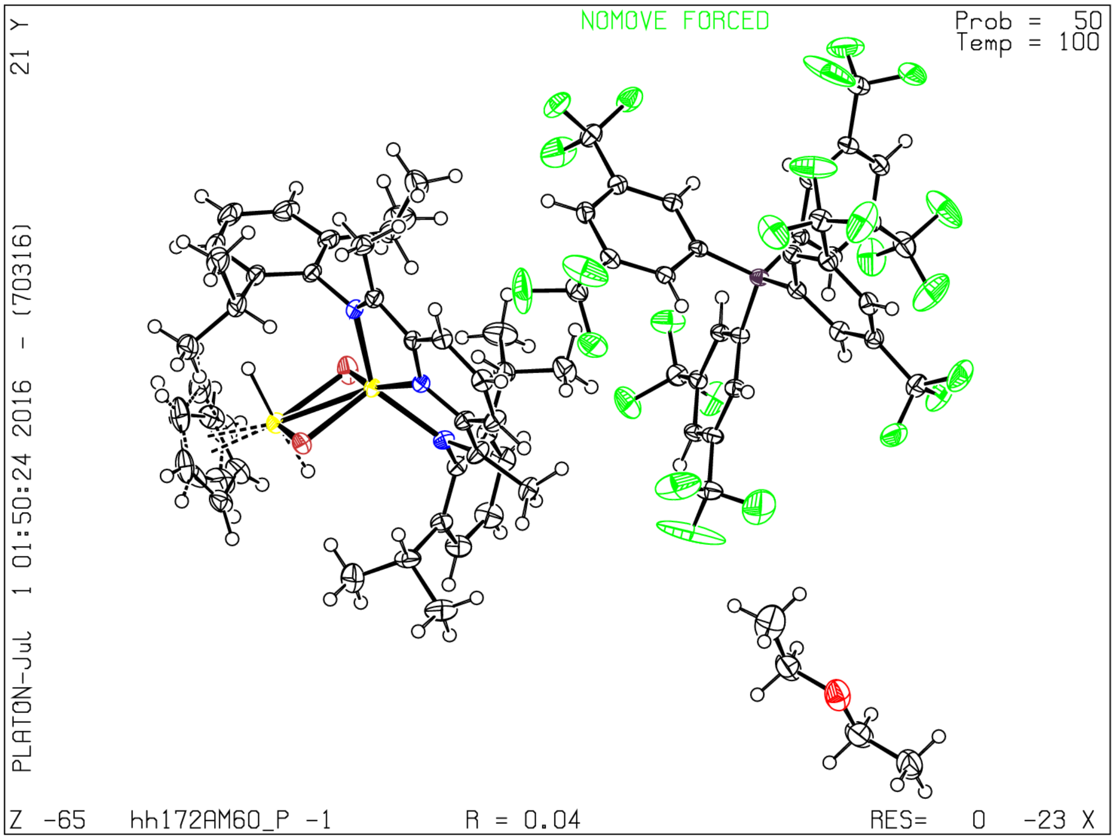

checkCIF/PLATON report

Structure factors have been supplied for datablock(s) hh166AM58_0m

THIS REPORT IS FOR GUIDANCE ONLY. IF USED AS PART OF A REVIEW PROCEDURE FOR PUBLICATION, IT SHOULD NOT REPLACE THE EXPERTISE OF AN EXPERIENCED CRYSTALLOGRAPHIC REFEREE.

No syntax errors found. CIF dictionary Interpreting this report

Datablock: hh166AM58_0m

```
Bond precision:  C-C = 0.0023 A          Wavelength=0.71073

Cell:           a=11.6274(6)       b=11.9639(6)     c=18.4816(10)
                alpha=75.9380(8)   beta=81.0945(8)  gamma=65.2264(7)
Temperature:    100 K
```

	Calculated	Reported
Volume	2260.3(2)	2260.3(2)
Space group	P -1	P -1
Hall group	-P 1	-P 1
Moiety formula	C39 H49 F3 Mo2 N3 O3 S3, C F3 O3 S	?
Sum formula	C40 H49 F6 Mo2 N3 O6 S4	C40 H49 F6 Mo2 N3 O6 S4
Mr	1101.94	1101.94
Dx,g cm-3	1.619	1.619
Z	2	2
Mu (mm-1)	0.812	0.812
F000	1120.0	1120.0
F000'	1114.61	
h,k,lmax	15,15,24	15,15,24
Nref	11215	11204
Tmin,Tmax	0.677,0.866	0.767,0.825
Tmin'	0.664	

```
Correction method= # Reported T Limits: Tmin=0.767 Tmax=0.825
AbsCorr = MULTI-SCAN

Data completeness= 0.999           Theta(max)= 28.282

R(reflections)= 0.0208( 10726)     wR2(reflections)= 0.0512( 11204)

S = 1.072                  Npar= 621
```

The following ALERTS were generated. Each ALERT has the format
test-name_ALERT_alert-type_alert-level.
Click on the hyperlinks for more details of the test.

Alert level C

PLAT220_ALERT_2_C	Non-Solvent Resd 1 Mo Ueq(max)/Ueq(min) Range	4.0 Ratio
PLAT222_ALERT_3_C	Non-Solvent Resd 1 H Uiso(max)/Uiso(min) Range	7.7 Ratio
PLAT601_ALERT_2_C	Structure Contains Solvent Accessible VOIDS of .	35 Ang3
PLAT911_ALERT_3_C	Missing # FCF Refl Between THmin & STh/L= 0.600	3 Report

---

Alert level G

PLAT002_ALERT_2_G	Number of Distance or Angle Restraints on AtSite	14 Note
PLAT003_ALERT_2_G	Number of Uiso or Uij Restrained non-H Atoms ...	10 Report
PLAT176_ALERT_4_G	The CIF-Embedded .res File Contains SADI Records	3 Report
PLAT177_ALERT_4_G	The CIF-Embedded .res File Contains DELU Records	1 Report
PLAT232_ALERT_2_G	Hirshfeld Test Diff (M-X) Mo1 -- S1 ..	6.6 s.u.
PLAT232_ALERT_2_G	Hirshfeld Test Diff (M-X) Mo2D -- S1 ..	14.3 s.u.
PLAT232_ALERT_2_G	Hirshfeld Test Diff (M-X) Mo2D -- S2 ..	17.0 s.u.
PLAT244_ALERT_4_G	Low 'Solvent' Ueq as Compared to Neighbors of	C1T Check
PLAT301_ALERT_3_G	Main Residue Disorder .... Percentage =	11 Note
PLAT432_ALERT_2_G	Short Inter X...Y Contact O1T .. C4 ..	2.92 Ang.
PLAT432_ALERT_2_G	Short Inter X...Y Contact O1T .. C3 ..	2.98 Ang.
PLAT434_ALERT_2_G	Short Inter HL..HL Contact F4T .. F4T ..	2.80 Ang.
PLAT860_ALERT_3_G	Number of Least-Squares Restraints	41 Note
PLAT910_ALERT_3_G	Missing # of FCF Reflection(s) Below Theta(Min)	2 Note
PLAT912_ALERT_4_G	Missing # of FCF Reflections Above STh/L= 0.600	7 Note
PLAT978_ALERT_2_G	Number C-C Bonds with Positive Residual Density	16 Note

---

0 **ALERT level A** = Most likely a serious problem - resolve or explain
0 **ALERT level B** = A potentially serious problem, consider carefully
4 **ALERT level C** = Check. Ensure it is not caused by an omission or oversight
16 **ALERT level G** = General information/check it is not something unexpected

0 ALERT type 1 CIF construction/syntax error, inconsistent or missing data
11 ALERT type 2 Indicator that the structure model may be wrong or deficient
5 ALERT type 3 Indicator that the structure quality may be low
4 ALERT type 4 Improvement, methodology, query or suggestion
0 ALERT type 5 Informative message, check

It is advisable to attempt to resolve as many as possible of the alerts in all categories. Often the minor alerts point to easily fixed oversights, errors and omissions in your CIF or refinement strategy, so attention to these fine details can be worthwhile. In order to resolve some of the more serious problems it may be necessary to carry out additional measurements or structure refinements. However, the purpose of your study may justify the reported deviations and the more serious of these should normally be commented upon in the discussion or experimental section of a paper or in the "special_details" fields of the CIF. checkCIF was carefully designed to identify outliers and unusual parameters, but every test has its limitations and alerts that are not important in a particular case may appear. Conversely, the absence of alerts does not guarantee there are no aspects of the results needing attention. It is up to the individual to critically assess their own results and, if necessary, seek expert advice.

Publication of your CIF in IUCr journals

A basic structural check has been run on your CIF. These basic checks will be run on all CIFs submitted for publication in IUCr journals (*Acta Crystallographica*, *Journal of Applied Crystallography*, *Journal of Synchrotron Radiation*); however, if you intend to submit to *Acta Crystallographica Section C* or *E* or *IUCrData*, you should make sure that full publication checks are run on the final version of your CIF prior to submission.

Publication of your CIF in other journals

Please refer to the *Notes for Authors* of the relevant journal for any special instructions relating to CIF submission.

PLATON version of 06/05/2016; check.def file version of 05/05/2016

Datablock hh166AM58_0m - ellipsoid plot

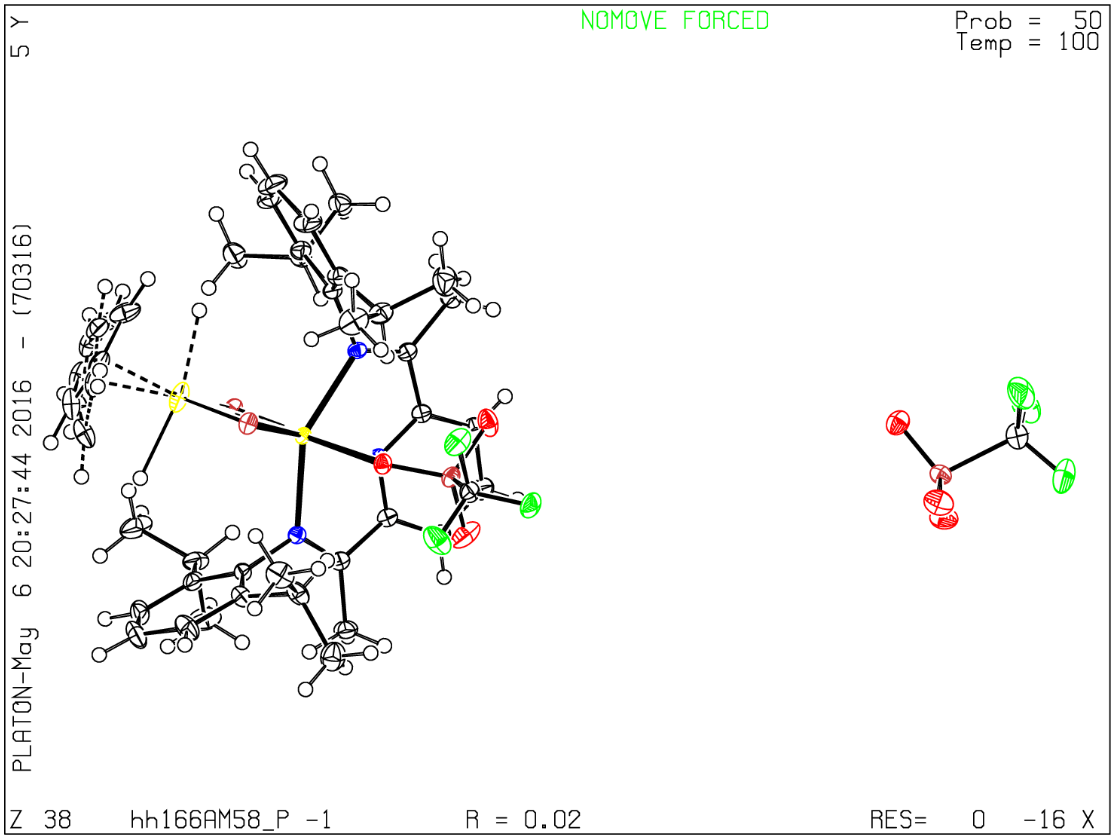

checkCIF/PLATON report

Structure factors have been supplied for datablock(s) hh75AM22_0m

THIS REPORT IS FOR GUIDANCE ONLY. IF USED AS PART OF A REVIEW PROCEDURE FOR PUBLICATION, IT SHOULD NOT REPLACE THE EXPERTISE OF AN EXPERIENCED CRYSTALLOGRAPHIC REFEREE.

No syntax errors found. CIF dictionary Interpreting this report

Datablock: hh75AM22_0m

```
Bond precision:  C-C = 0.0018 A          Wavelength=0.71073

Cell:          a=12.1696(2)       b=14.8304(3)      c=16.7360(3)
               alpha=64.6153(5)   beta=70.7927(5)   gamma=73.5133(5)
Temperature:   100 K
```

	Calculated	Reported
Volume	2540.65(8)	2540.65(8)
Space group	P -1	P -1
Hall group	-P 1	-P 1
Moiety formula	C42 H54 Mo2 N5 S2, 2(B F4), 2(C2 H3 N)	?
Sum formula	C46 H60 B2 F8 Mo2 N7 S2	C46 H60 B2 F8 Mo2 N7 S2
Mr	1140.72	1140.63
Dx,g cm-3	1.491	1.491
Z	2	2
Mu (mm-1)	0.645	0.645
F000	1166.1	1166.0
F000'	1160.17	
h,k,lmax	17,20,23	17,20,23
Nref	14871	14859
Tmin,Tmax	0.749,0.877	0.703,0.747
Tmin'	0.734	

```
Correction method= # Reported T Limits: Tmin=0.703 Tmax=0.747
AbsCorr = MULTI-SCAN

Data completeness= 0.999          Theta(max)= 30.033

R(reflections)= 0.0193( 13854)    wR2(reflections)= 0.0493( 14859)

S = 1.029                   Npar= 693
```

The following ALERTS were generated. Each ALERT has the format
test-name_ALERT_alert-type_alert-level.
Click on the hyperlinks for more details of the test.

Alert level C

```
PLAT244_ALERT_4_C Low   'Solvent' Ueq as Compared to Neighbors of          B2 Check
PLAT731_ALERT_1_C Bond    Calc   2.8148(4), Rep 2.81475(13) ......          3 su-Rat
              MO1  -MO2     1.555   1.555                #        23
PLAT732_ALERT_1_C Angle   Calc   102.60(2), Rep   102.60(1) ......       2.22 su-Rat
              S1   -MO1  -S2      1.555   1.555   1.555      #        39
PLAT732_ALERT_1_C Angle   Calc   105.99(2), Rep   105.99(1) ......       2.22 su-Rat
              S1   -MO2  -S2      1.555   1.555   1.555      #       208
PLAT911_ALERT_3_C Missing # FCF Refl Between THmin & STh/L=  0.600          4 Report
```

Alert level G

```
PLAT002_ALERT_2_G Number of Distance or Angle Restraints on AtSite         15 Note
PLAT003_ALERT_2_G Number of Uiso or Uij Restrained non-H Atoms ...         15 Report
PLAT171_ALERT_4_G The CIF-Embedded .res File Contains EADP Records          3 Report
PLAT172_ALERT_4_G The CIF-Embedded .res File Contains DFIX Records          3 Report
PLAT176_ALERT_4_G The CIF-Embedded .res File Contains SADI Records          3 Report
PLAT177_ALERT_4_G The CIF-Embedded .res File Contains DELU Records          1 Report
PLAT230_ALERT_2_G Hirshfeld Test Diff for    C2S    --  C3S     ..        5.4 su
PLAT230_ALERT_2_G Hirshfeld Test Diff for    C4S    --  C5S     ..        5.9 su
PLAT232_ALERT_2_G Hirshfeld Test Diff (M-X)  Mo1    --  S1      ..       10.5 su
PLAT232_ALERT_2_G Hirshfeld Test Diff (M-X)  Mo1    --  S2      ..       12.3 su
PLAT232_ALERT_2_G Hirshfeld Test Diff (M-X)  Mo1    --  N3S     ..       12.3 su
PLAT232_ALERT_2_G Hirshfeld Test Diff (M-X)  Mo2    --  S2      ..        9.5 su
PLAT232_ALERT_2_G Hirshfeld Test Diff (M-X)  Mo2    --  N1S     ..       14.5 su
PLAT300_ALERT_4_G Atom Site Occupancy of >F1     is Constrained at      0.532 Check
PLAT300_ALERT_4_G Atom Site Occupancy of >F2     is Constrained at      0.532 Check
PLAT300_ALERT_4_G Atom Site Occupancy of >F3     is Constrained at      0.532 Check
PLAT300_ALERT_4_G Atom Site Occupancy of >F4     is Constrained at      0.532 Check
PLAT300_ALERT_4_G Atom Site Occupancy of >B1     is Constrained at      0.532 Check
PLAT300_ALERT_4_G Atom Site Occupancy of <F1D    is Constrained at      0.364 Check
PLAT300_ALERT_4_G Atom Site Occupancy of <F2D    is Constrained at      0.364 Check
PLAT300_ALERT_4_G Atom Site Occupancy of <F3D    is Constrained at      0.364 Check
PLAT300_ALERT_4_G Atom Site Occupancy of <F4D    is Constrained at      0.364 Check
PLAT300_ALERT_4_G Atom Site Occupancy of <B1D    is Constrained at      0.364 Check
PLAT300_ALERT_4_G Atom Site Occupancy of <F1E    is Constrained at      0.105 Check
PLAT300_ALERT_4_G Atom Site Occupancy of <F2E    is Constrained at      0.105 Check
PLAT300_ALERT_4_G Atom Site Occupancy of <F3E    is Constrained at      0.105 Check
PLAT300_ALERT_4_G Atom Site Occupancy of <F4E    is Constrained at      0.105 Check
PLAT300_ALERT_4_G Atom Site Occupancy of <B1E    is Constrained at      0.105 Check
PLAT302_ALERT_4_G Anion/Solvent Disorder ........... Percentage =          31 Note
PLAT304_ALERT_4_G Non-Integer Number of Atoms (   2.66) in Resd. #          2 Check
PLAT304_ALERT_4_G Non-Integer Number of Atoms (   1.82) in Resd. #          4 Check
PLAT304_ALERT_4_G Non-Integer Number of Atoms (   0.52) in Resd. #          5 Check
PLAT380_ALERT_4_G Incorrectly? Oriented X(sp2)-Methyl Moiety .....        C21 Check
PLAT860_ALERT_3_G Number of Least-Squares Restraints ............          88 Note
PLAT910_ALERT_3_G Missing # of FCF Reflection(s) Below Th(Min) ...          1 Report
PLAT912_ALERT_4_G Missing # of FCF Reflections Above STh/L=  0.600          8 Note
```

---

0 **ALERT level A** = Most likely a serious problem - resolve or explain
0 **ALERT level B** = A potentially serious problem, consider carefully
5 **ALERT level C** = Check. Ensure it is not caused by an omission or oversight
36 **ALERT level G** = General information/check it is not something unexpected

3 ALERT type 1 CIF construction/syntax error, inconsistent or missing data

```
 9 ALERT type 2 Indicator that the structure model may be wrong or deficient
 3 ALERT type 3 Indicator that the structure quality may be low
26 ALERT type 4 Improvement, methodology, query or suggestion
 0 ALERT type 5 Informative message, check
```

It is advisable to attempt to resolve as many as possible of the alerts in all categories. Often the minor alerts point to easily fixed oversights, errors and omissions in your CIF or refinement strategy, so attention to these fine details can be worthwhile. In order to resolve some of the more serious problems it may be necessary to carry out additional measurements or structure refinements. However, the purpose of your study may justify the reported deviations and the more serious of these should normally be commented upon in the discussion or experimental section of a paper or in the "special_details" fields of the CIF. checkCIF was carefully designed to identify outliers and unusual parameters, but every test has its limitations and alerts that are not important in a particular case may appear. Conversely, the absence of alerts does not guarantee there are no aspects of the results needing attention. It is up to the individual to critically assess their own results and, if necessary, seek expert advice.

Publication of your CIF in IUCr journals

A basic structural check has been run on your CIF. These basic checks will be run on all CIFs submitted for publication in IUCr journals (*Acta Crystallographica*, *Journal of Applied Crystallography*, *Journal of Synchrotron Radiation*); however, if you intend to submit to *Acta Crystallographica Section C* or *E*, you should make sure that full publication checks are run on the final version of your CIF prior to submission.

Publication of your CIF in other journals

Please refer to the *Notes for Authors* of the relevant journal for any special instructions relating to CIF submission.

PLATON version of 19/12/2014; check.def file version of 19/12/2014

Datablock hh75AM22_0m - ellipsoid plot

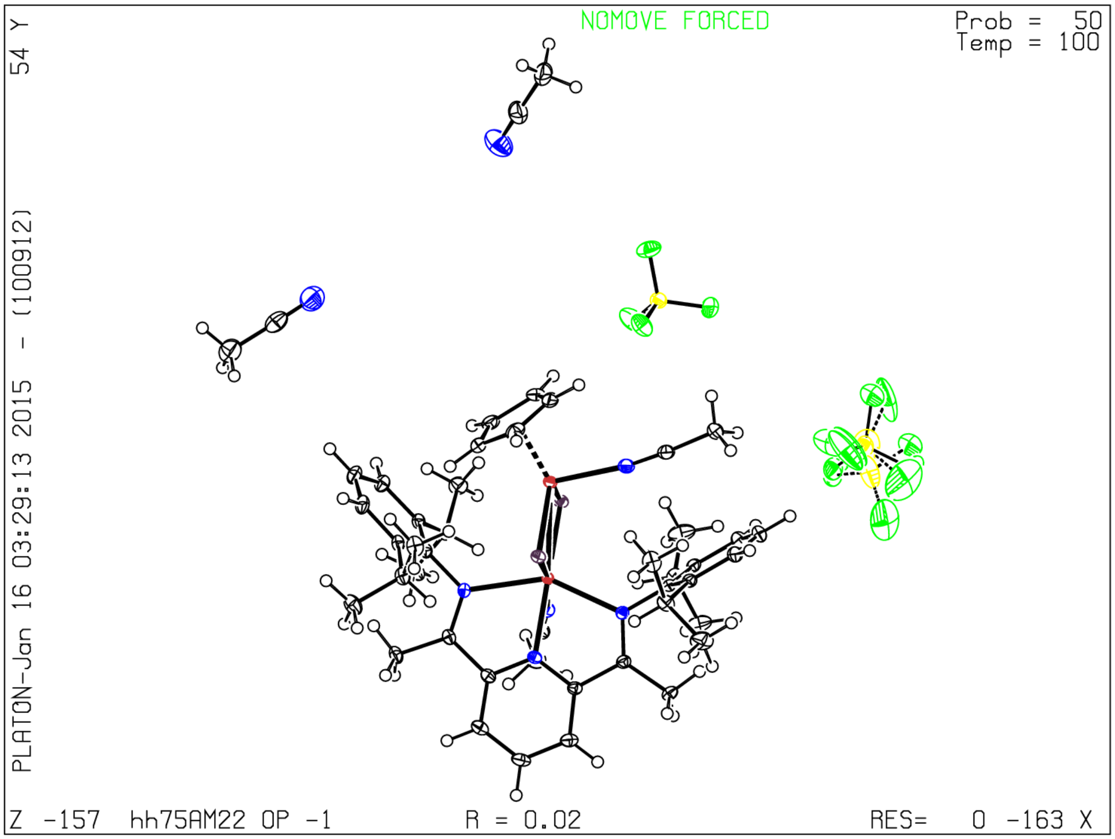

Supplement: Supplementary file 1 [file molecules-25-03090-s001.zip › checkCIF.pdf]
