# Supplementary material for: Assembly and Redox-Rich Hydride Chemistry of an Asymmetric Mo2S2 Platform
Source: Molecules. 2020 Jul 7;25(13):3090. doi: 10.3390/molecules25133090 (PMC7411697; doi:10.3390/molecules25133090)
Supplement: Supplementary file 1 [file molecules-25-03090-s001.zip › SI.pdf]

Supporting Information for:

Assembly and Redox-Rich Hydride Chemistry of an Asymmetric Mo_2S_2 Platform

Alex McSkimming, Jordan W. Taylor and W. Hill Harman*

Department of Chemistry, University of California, Riverside, California, USA

Contents

Additional XRD Data	3
NMR Data	4
FT-IR Data	5
Additional NMR data	7
Additional Electrochemistry Data	8
Calculated Coordinates	9

Additional XRD Data

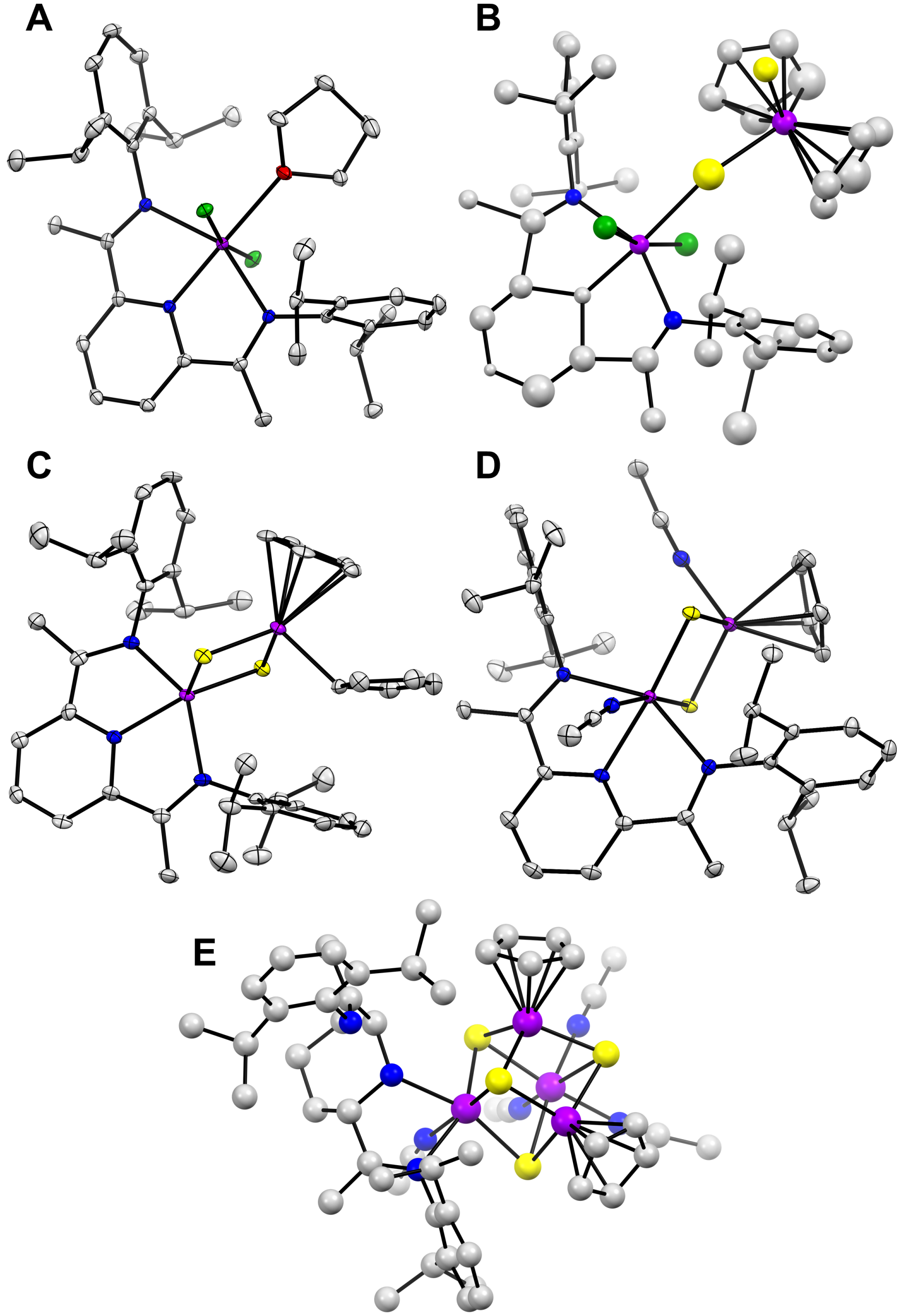

Figure S1. Thermal ellipsoid plots (50%) of (A) **1**, (C) **3**, (D) **5** and XRD connectivity assignments for (B) **2** and (E) **6**. Magenta, yellow, blue, orange, green and grey ellipsoids represent Mo, S, N, Na, F and C, respectively. Hydrogen atoms, solvent molecules and counter-ions are omitted for clarity.

NMR Data

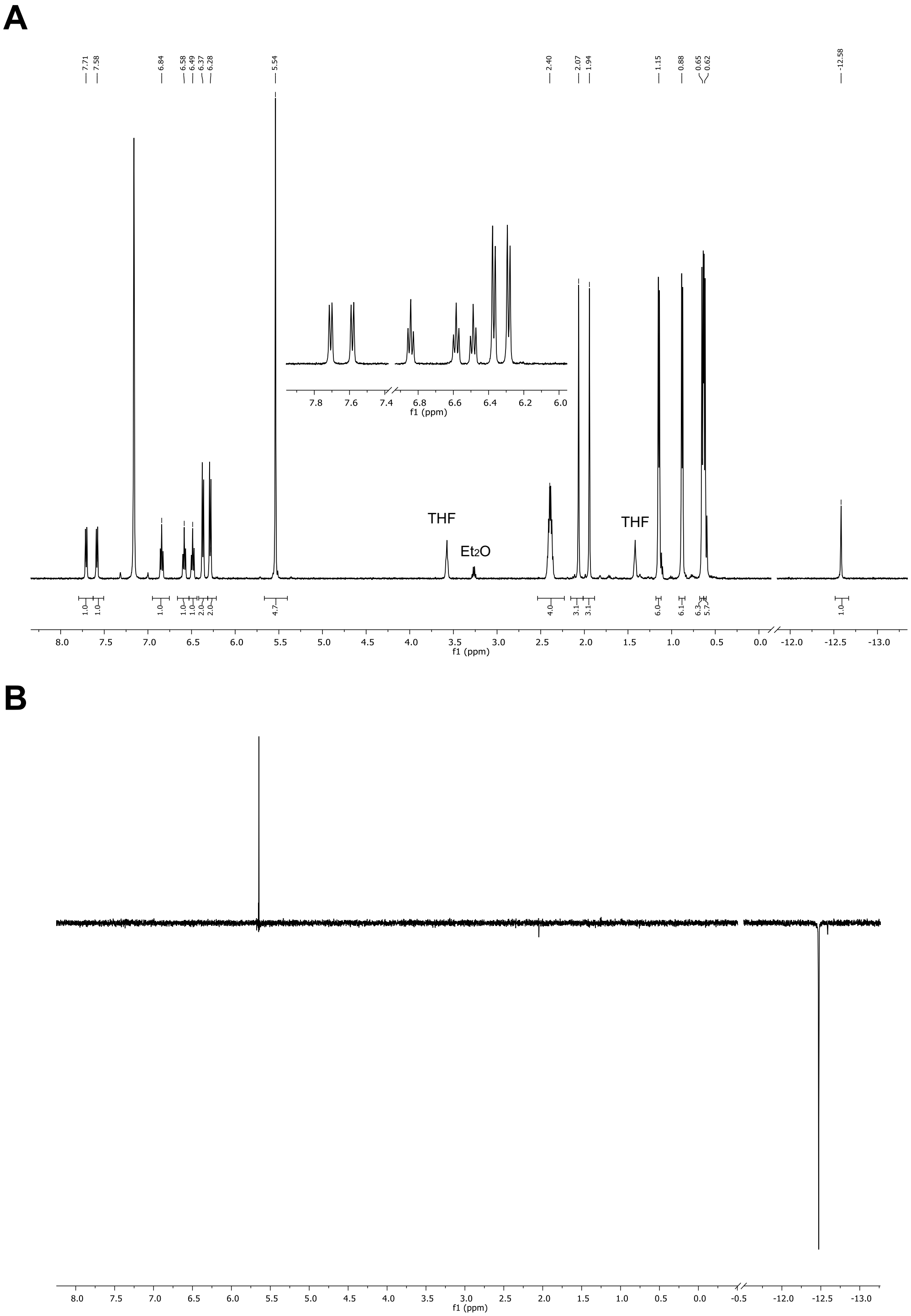

Figure S2. (A) ^{1}H NMR spectrum of **4** recorded in C_6D_6 at 400 MHz; aromatic region inset. (B) 1D-^{1}H-^{1}H NOESY spectrum recorded for **4** with selective irradiation of the hydride resonance.

FT-IR Data

A

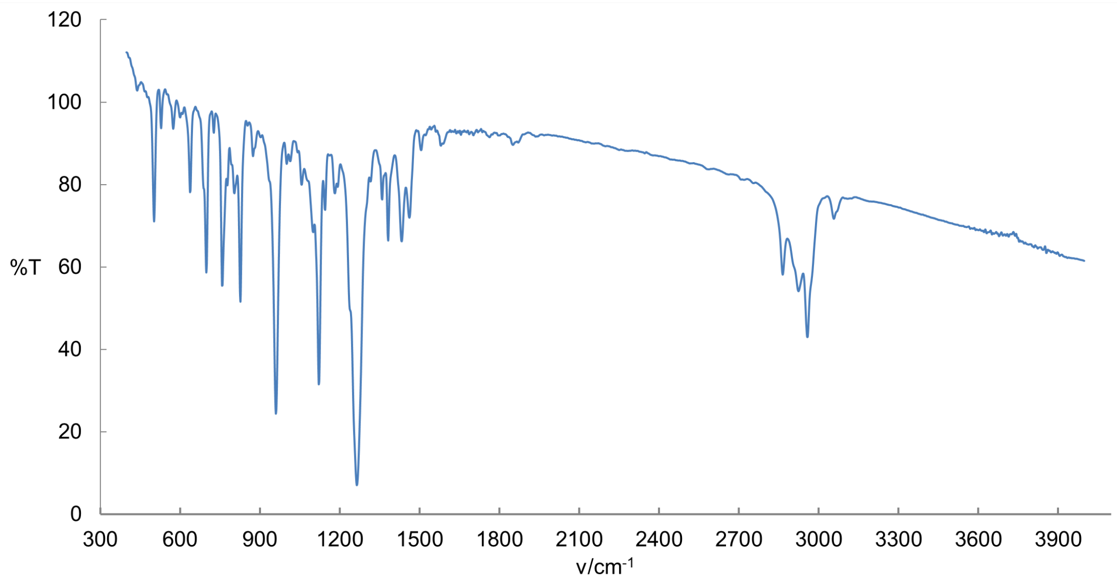

B

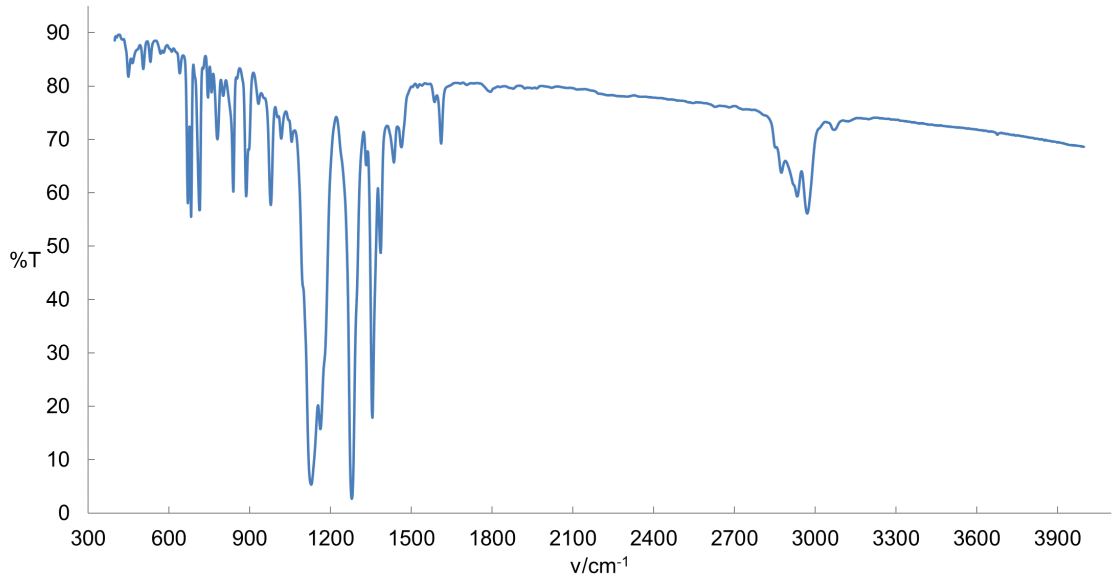

Figure S3. FT-IR spectra of (A) **4** and (B) [**4**][BAr$^{F}{}_{4}$]

A

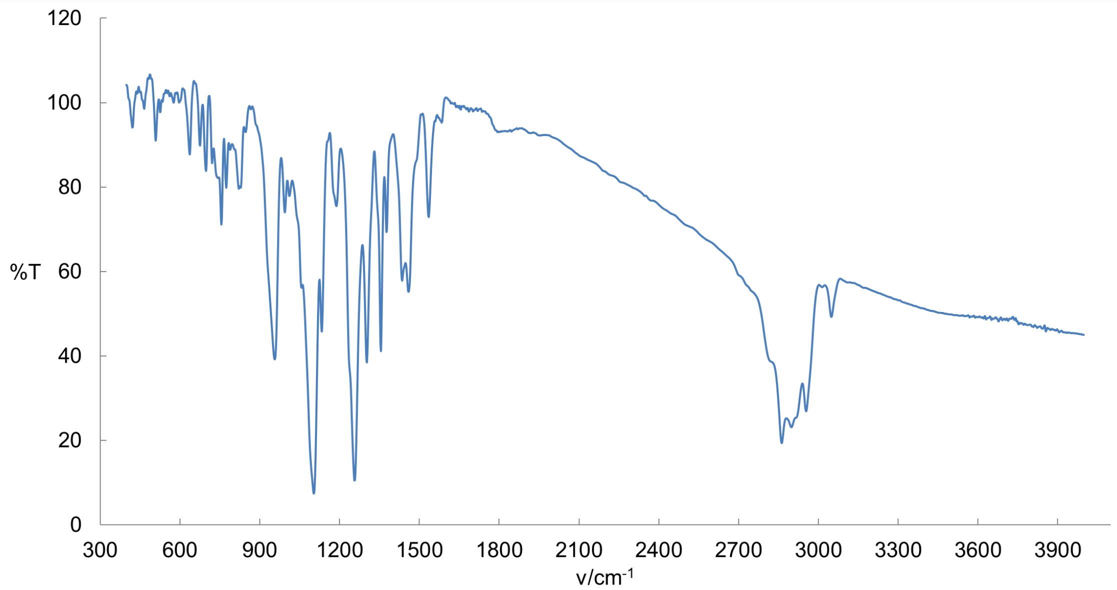

B

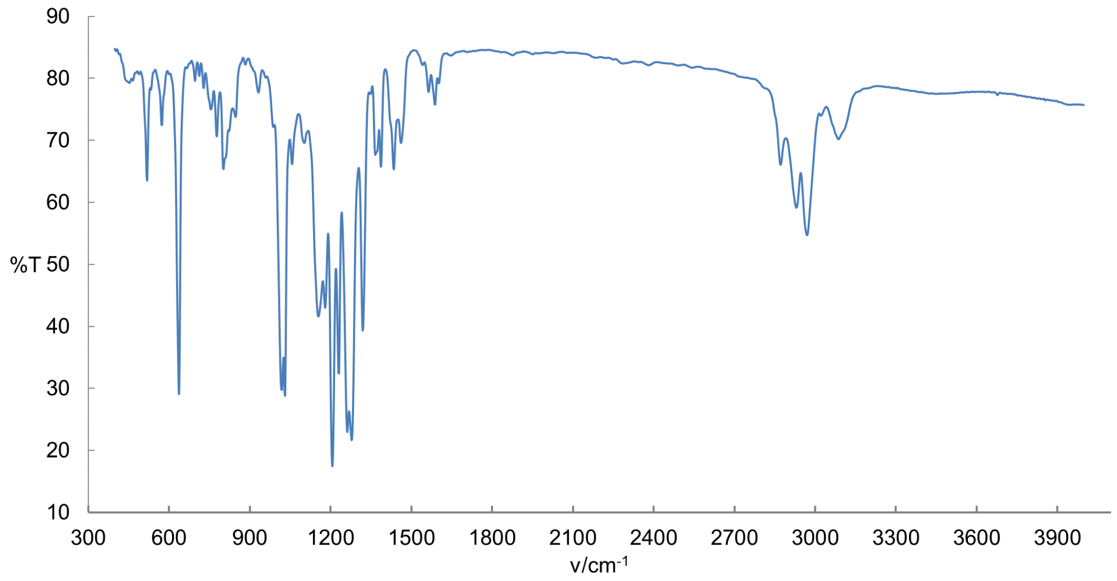

Figure S4. FT-IR spectra of (A) [**4**][Na(crypt-222)] and (B) [**4**-OTf][OTf]

Additional NMR Data

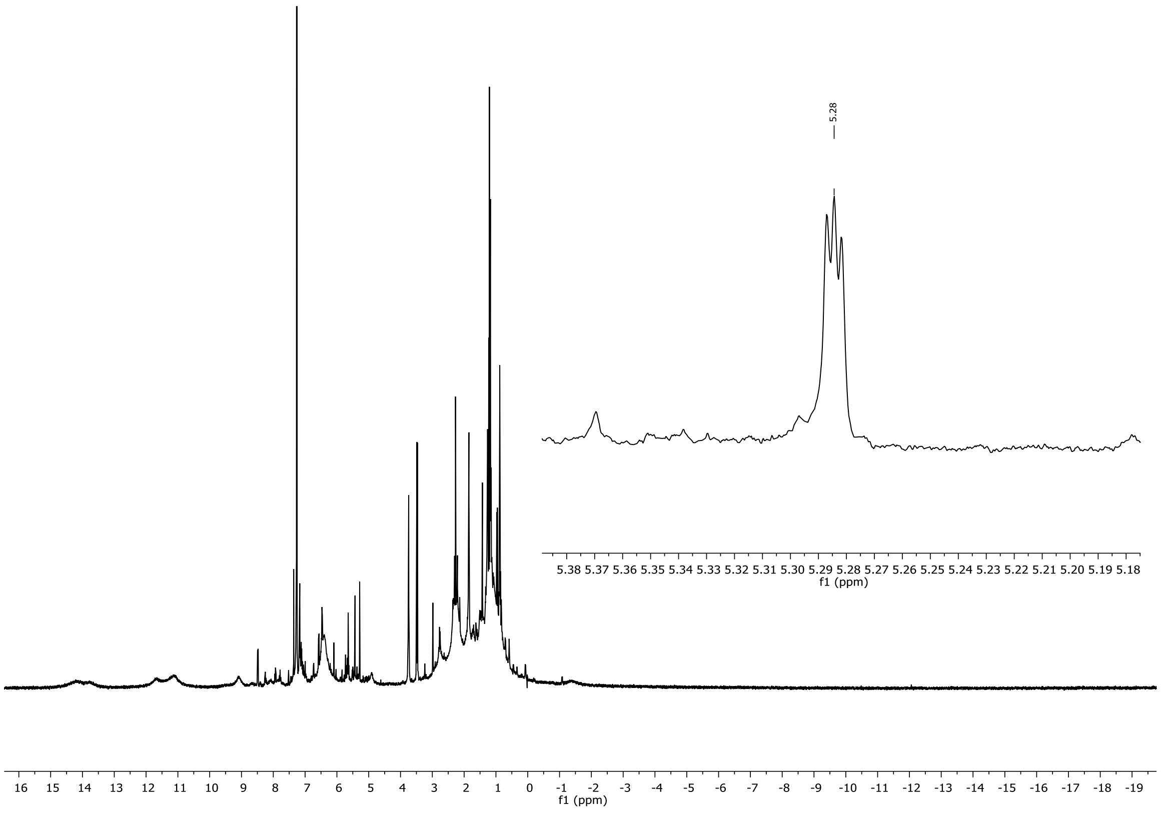

Figure S5. ^{1}H NMR spectrum of **4** in $CDCl_3$ recorded after ~1h. The inset highlights the peak corresponding to CD_2Cl_2.

Additional Electrochemistry Data

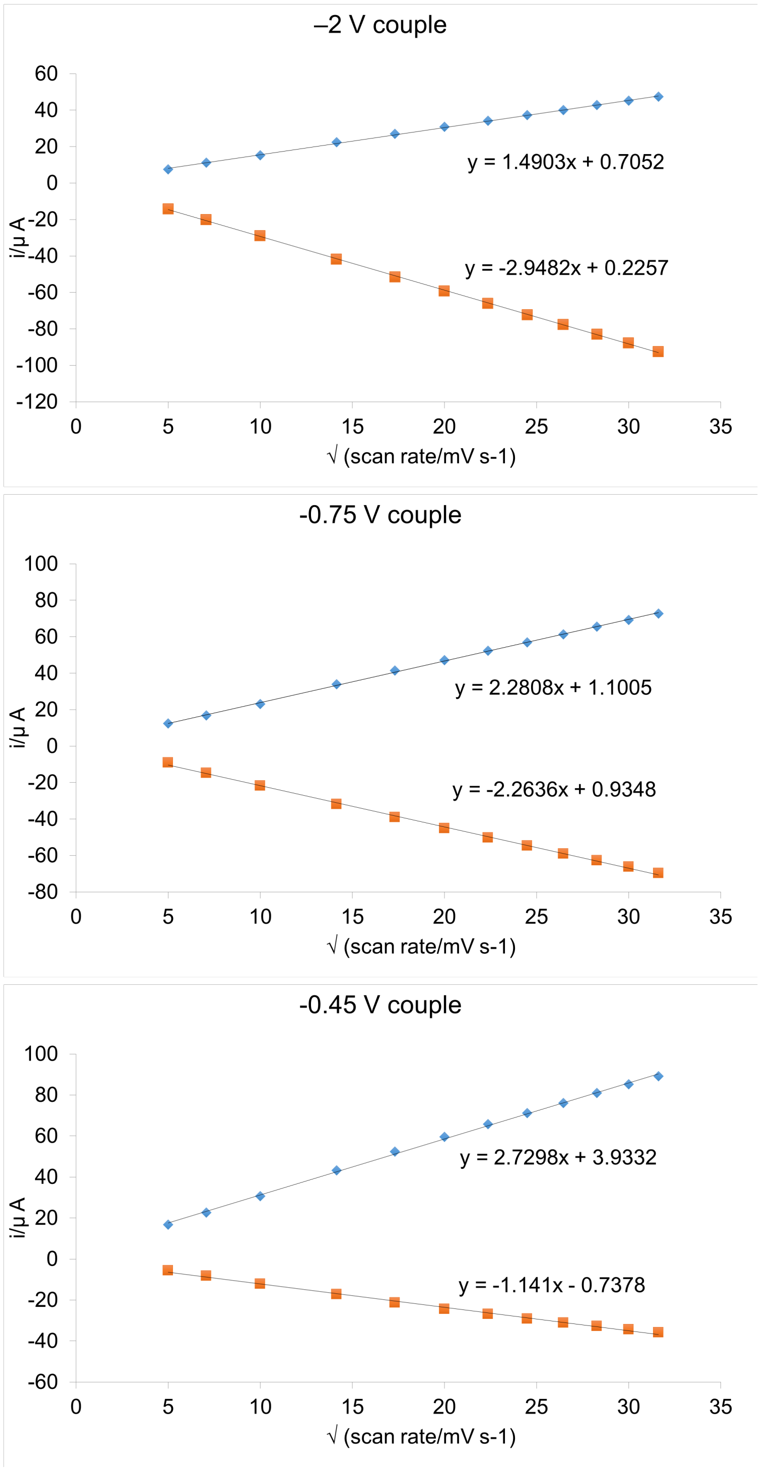

Figure S6. Associated Randles-Sevcik plots for the CV of **4** (shown in Figure 1).

Calculated Coordinates

4

Mo	2.38168780478325	2.88944353204584	3.60398673640427
S	1.16455743724895	3.49091118498181	5.40134218367252
S	0.80164569974237	2.67557524042107	1.95862627788800
N	3.34862844096988	4.61087995053686	2.87935950300953
C	4.62901591558924	4.88485775423739	3.21512912515302
N	4.38299296980569	2.76636632752836	4.12552224390736
C	5.25276657389370	3.83238733915413	3.90727773406873
C	6.57640716222714	3.78133542533950	4.34133869055846
H	7.21333368031292	4.65179352728642	4.17716629695100
C	7.07970600058751	2.62836130815716	4.92212835593077
H	8.11387583108979	2.58187718021346	5.26167463158140
C	6.26349406454660	1.49430534523679	4.97757525982448
H	6.65611555880458	0.53579302693959	5.31901778592095
C	4.94388425329363	1.56383638270885	4.54378108105776
C	4.04317448113332	0.48409342006709	4.42006394066842
N	2.86514382481358	0.85497220097351	3.89071988006955
Mo	-0.40970644804705	3.29286926921937	3.74126577109843
H	-0.72001293315727	1.67809849361889	4.09793462387693
C	-2.48854947368981	3.33550425641230	5.05760298550578
H	-2.59731791923059	2.74722576661529	5.96359171898779
C	-1.99058009079070	4.63896460441782	4.97665269551207
H	-1.64839161293946	5.25550524260045	5.80202141705736
C	-1.93466966026054	4.98479550536598	3.59704019833319
H	-1.59498216755133	5.93797775623374	3.19777321606903
C	-2.44773080226646	3.91574689933765	2.84792160576484
H	-2.59001441674880	3.89206005990758	1.77212254794232
C	-2.75523366596550	2.89324943287264	3.75040592933818
H	-3.16284387448094	1.92208793802242	3.48619607023471
H	4.24795505239257	-0.53443203414504	4.74800846897737
H	2.15156534165413	0.13380778671699	3.78689031474872
H	2.83617685294495	5.34031357188581	2.38567339781836
H	5.09478711929460	5.84641430509025	3.00246231206865

[**4**]$^{+}$

Mo	-0.087664	9.595674	4.523336
N	-0.545800	7.684646	5.242330
N	1.105241	9.245205	6.247736
C	-0.100167	7.267571	6.440737
C	0.846874	8.172559	7.040166
C	1.559849	7.963842	8.217267
H	1.326938	7.103556	8.844181
C	2.594705	8.827556	8.541680
H	3.161644	8.681309	9.460325
C	2.954541	9.832716	7.652495

H	3.828135	10.458758	7.831518
C	2.205851	10.006837	6.497171
C	2.504614	10.894354	5.402624
N	1.662406	10.800108	4.392828
S	-1.567403	11.013619	5.435480
S	-0.765019	9.437420	2.339291
Mo	-2.186023	10.902932	3.244888
H	-1.096153	12.122763	2.904861
C	-3.317550	11.660819	1.345093
H	-2.891020	11.516516	0.357210
C	-4.159591	10.758815	2.028048
H	-4.508160	9.799900	1.655268
C	-4.488725	11.345938	3.279844
H	-5.126409	10.906604	4.042351
C	-3.854242	12.595390	3.358485
H	-3.905733	13.287371	4.193443
C	-3.123925	12.782215	2.175568
H	-2.540022	13.661473	1.922979
H	3.345838	11.588568	5.405132
H	1.819806	11.422533	3.598025
H	-0.422216	6.330086	6.892993
H	-1.209433	7.068287	4.770360

[**4**]$^{-}$

Mo	12.883340	4.209770	11.985154
S	14.877986	4.717100	10.959846
S	11.955902	2.633320	10.578585
N	11.785582	5.931733	11.274074
N	12.114851	5.108091	13.632633
N	13.443006	2.970237	13.626699
C	11.164044	6.700134	12.201481
C	11.319687	6.265016	13.525735
C	10.784963	6.867779	14.661907
H	10.168446	7.761276	14.540071
C	11.038503	6.342079	15.931233
H	10.622195	6.822277	16.818462
C	11.839653	5.200393	16.058241
H	12.054020	4.769649	17.038529
C	12.355140	4.575982	14.919766
C	13.102642	3.384045	14.881466
Mo	13.904989	3.191098	9.555767
H	14.838155	1.899195	10.143288
C	15.377333	3.408753	7.728023
H	16.376489	3.806057	7.876103
C	14.214431	4.147485	7.499719
H	14.150636	5.229451	7.421130
C	13.129522	3.232219	7.388366
H	12.090506	3.499340	7.215642
C	13.621120	1.960180	7.550121
H	13.032090	1.047156	7.544190

C	15.000608	2.034377	7.780391
H	15.668227	1.198605	7.961818
H	13.936569	2.077472	13.582756
H	13.357140	2.811630	15.775414
H	10.578122	7.582849	11.935935
H	11.660390	6.231877	10.306326

[**4**-OTf]$^+$

Mo	2.59092164813932	1.48752623521056	4.83447001609312
S	1.70322028981881	1.53617752794889	6.91200782112357
S	0.91340668875520	0.63786449739540	3.51581052862545
N	2.33916605474956	3.45739418097630	3.95367324560120
N	3.98833314183592	1.66765756927565	3.17978435588744
N	3.74271108306778	-0.35061800834437	4.70767640363625
C	3.11947826516867	3.84607749533789	2.99359079060788
C	4.10531714048157	2.84832731454537	2.54353952333850
C	5.10156286963125	3.05850886648306	1.59748082424384
H	5.19239577763868	4.02734151233705	1.10880926576130
C	5.98888384895493	2.02705277082894	1.32225321375686
H	6.77867671406400	2.17248706912273	0.58584659173872
C	5.89975717957020	0.83260497546318	2.01770828977040
H	6.62002224587228	0.03051507209641	1.86491810047713
C	4.89394026359692	0.69505832348233	2.96675035609672
C	4.72510772967362	-0.45277244988334	3.86537782370928
Mo	0.05824556672911	0.68254570686917	5.61693058707029
H	0.55692168566462	-0.86876967839892	6.00555321258498
C	-1.60763687654650	-0.63861322146200	6.59039266097539
H	-1.41482065260415	-1.59555166443398	7.06516287598555
C	-1.96368744669290	-0.44895035093311	5.23421915826894
H	-2.05859592540113	-1.22774112974347	4.48407094256188
C	-2.14792218436243	0.92882307728207	5.03268335976901
H	-2.42922814096097	1.40843823468361	4.09966843702540
C	-1.88343756592155	1.58806258970768	6.26287721616440
H	-1.94804284406277	2.65880844033956	6.44074913129090
C	-1.57736261340135	0.61322607037344	7.22619395977019
H	-1.34152321774988	0.79554100326734	8.27015328252314
S	5.71132555663434	2.97237759327150	5.46159825901323
O	4.41890297078331	2.37728202482788	5.84616260537852
O	6.65115898964120	2.02470578272907	4.94713635100331
O	5.58669327997862	4.22804239428989	4.77746594156332
C	6.38710382138194	3.43176963824336	7.09318614802070
F	6.52971501540821	2.37126568329189	7.87928441112970
F	7.58751818798091	3.99174278372582	6.93461821113130
F	5.60721879332305	4.30972082807484	7.71702107347235
H	3.59387720409353	-1.13458644084377	5.34776410732243
H	5.37134353878063	-1.33109668790769	3.81123550214038
H	1.66828756099150	4.14523603966294	4.30330037141592
H	3.07052735529389	4.84518233080678	2.55675704395101
